# SG1002 and Catenated Divalent Organic Sulfur Compounds as Promising Hydrogen Sulfide Prodrugs

**DOI:** 10.1089/ars.2020.8060

**Published:** 2020-10-08

**Authors:** Gabriel Gojon, Guillermo A. Morales

**Affiliations:** Sulfagenix, Inc., Tucson, Arizona, USA.

**Keywords:** SG1002, TC-2153, H_2_S prodrug, hydrogen sulfide, polysulfides

## Abstract

***Significance:*** Sulfur has a critical role in protein structure/function and redox status/signaling in all living organisms. Although hydrogen sulfide (H_2_S) and sulfane sulfur (SS) are now recognized as central players in physiology and pathophysiology, the full scope and depth of sulfur metabolome's impact on human health and healthy longevity has been vastly underestimated and is only starting to be grasped. Since many pathological conditions have been related to abnormally low levels of H_2_S/SS in blood and/or tissues, and are amenable to treatment by H_2_S supplementation, development of safe and efficacious H_2_S donors deserves to be undertaken with a sense of urgency; these prodrugs also hold the promise of becoming widely used for disease prevention and as antiaging agents.

***Recent Advances:*** Supramolecular tuning of the properties of well-known molecules comprising chains of sulfur atoms (diallyl trisulfide [DATS], S_8_) was shown to lead to improved donors such as DATS-loaded polymeric nanoparticles and SG1002. Encouraging results in animal models have been obtained with SG1002 in heart failure, atherosclerosis, ischemic damage, and Duchenne muscular dystrophy; with TC-2153 in Alzheimer's disease, schizophrenia, age-related memory decline, fragile X syndrome, and cocaine addiction; and with DATS in brain, colon, gastric, and breast cancer.

***Critical Issues:*** Mode-of-action studies on allyl polysulfides, benzyl polysulfides, ajoene, and 12 ring-substituted organic disulfides and thiosulfonates led several groups of researchers to conclude that the anticancer effect of these compounds is not mediated by H_2_S and is only modulated by reactive oxygen species, and that their central model of action is selective protein S-thiolation.

***Future Directions:*** SG1002 is likely to emerge as the H_2_S donor of choice for acquiring knowledge on this gasotransmitter's effects in animal models, on account of its unique ability to efficiently generate H_2_S without byproducts and in a slow and sustained mode that is dose independent and enzyme independent. Efficient tuning of H_2_S donation characteristics of DATS, dibenzyl trisulfide, and other hydrophobic H_2_S prodrugs for both oral and parenteral administration will be achieved not only by conventional structural modification of a lead molecule but also through the new “supramolecular tuning” paradigm.

## Introduction

**FIG. 1.**
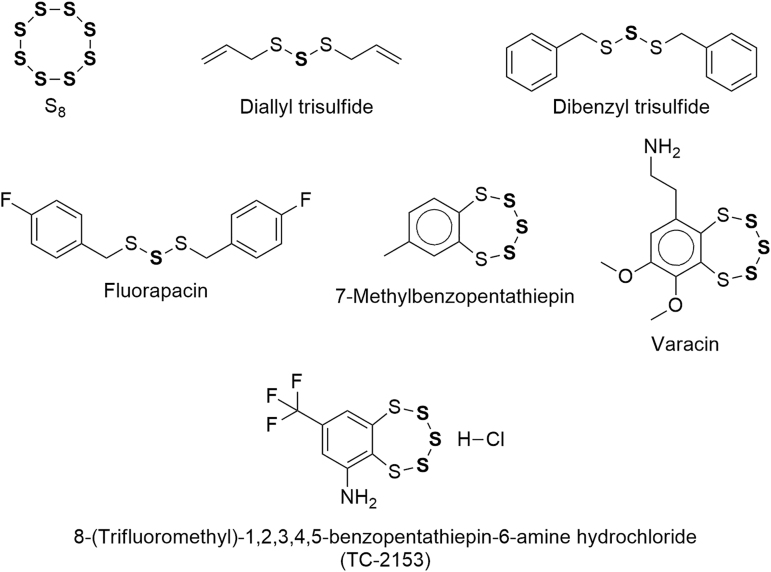
**Molecules containing sulfane sulfur (SS atoms in bold).** SS, sulfane sulfur.

**APPENDIX FIG. A1.**
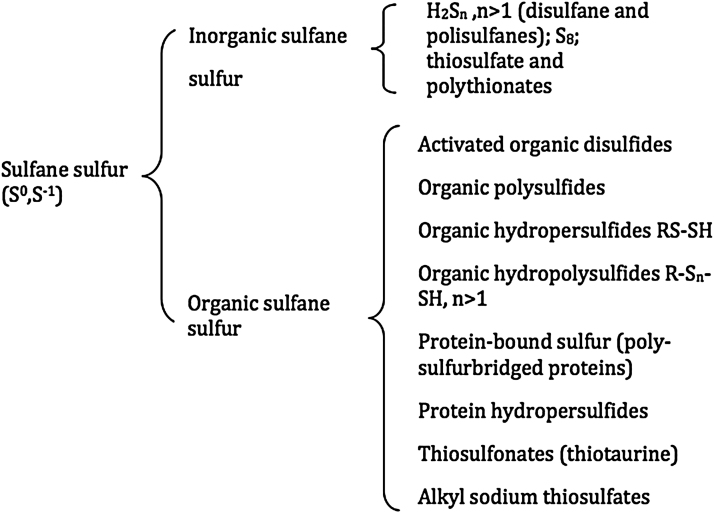
SS classification. Reprinted and modified with permission from Predmore *et al.* (202). Copyright 2012 Mary Ann Liebert, Inc., publishers.

**APPENDIX FIG. A2.**
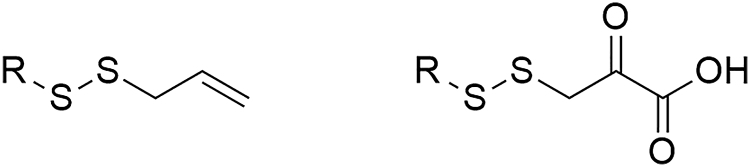
Activated organic disulfides. Adapted with permission from Predmore *et al.* (202). Copyright 2012 Mary Ann Liebert, Inc., publishers.

The present review focuses on a handful of catenated divalent sulfur molecules of low toxicity that contain sulfane sulfur (SS), are therefore able to release hydrogen sulfide (H_2_S) *via* chemical or enzymatic reduction, and have—at least in principle—the potential to be developed into H_2_S prodrugs. Since these fascinating molecules have been intensively studied by many generations of researchers in several fields of science, we will not be able to present all of their findings, but only the more salient ones and are always striving to discover previously unrecognized connections, to keep a critical stance, and to anticipate future developments. The structural formulas of these molecules are shown in Figure 1. For a background discussion on sulfane sulfur and hydrogen sulfide see the Appendix and Appendix Figures A1 and A2.

## Prodrug-Based H_2_S Supplementation Is a Valid Therapeutic Strategy

The homeostasis of mammals is maintained through the agency of a plethora of signaling chemical species that regulate the function of cells, tissues, and organs; among them, only NO, CO, and H_2_S—the so-called gasotransmitters—are endogenous diatomic or triatomic molecules that are capable of freely diffusing across cell membranes (171). These three small molecules play critical roles in both health and disease, as evidenced by the fact that most cells in our body are endowed with the enzymes required to produce them.

Of note, the roles of these small signaling molecules present some overlap: They usually operate in a concerted and cooperative manner, and significant alterations in tissue concentration of any one of them either has detrimental physiological consequences or reflects a disease state. However, many experimental results support the notion that limited bioavailability of H_2_S, NO, or CO may be counteracted by exogenously supplied H_2_S—partially through its action on endothelial nitric oxide synthase (242) and nuclear erythroid 2-related factor 2 (Nrf2) (96), respectively, but also by other means (18).

H_2_S, through SS and transcription factor Nrf2 (96, 247), is capable of transactivating more than 200 cytoprotective genes, thereby upregulating transcription of multiple antioxidant enzymes, phase II detoxifying enzymes, enzymes that catalyze the synthesis and regeneration of GSH, and enzymes in charge of regulating NADPH regeneration, mitochondrial bioenergetics, and lipid metabolism. Importantly, heme oxygenase-1 (HO-1) is the primary source of gasotransmitter CO. Moreover, it has been shown that H_2_S activation of signal transducer and activator of transcription 3 (STAT3) (163) induces the transcription of additional cytoprotective proteins, including heat shock proteins such as Hsp90, and that H_2_S inhibits Nf-kappa-beta (214), which is upregulated in many diseases related to inflammation—including cancer.

There are several reasons, in addition to the ones just mentioned, that strongly suggest assigning the highest priority to the development of H_2_S prodrugs; they will be presented in this and the next section.

### H_2_S-poor diseases and other H_2_S-treatable pathologies

Many correlations have been established between low levels of H_2_S in blood or tissue and the onset of disease states related to oxidative cell damage, and/or chronic inflammation (150), and/or immune dysfunction (79, 175), and/or endoplasmic reticulum (ER) stress (113, 274), and/or dysregulation of mitochondrial bioenergetics (244), and/or hyperproliferation of cells or viruses (12); such correlations suggest the existence of causal links that are being vigorously scrutinized. Moreover, in some instances, an inverse relationship between disease progression and H_2_S level in blood and/or tissues has been established (54, 110, 136, 200).

Pathological conditions associated with so-called “H_2_S-poor” disease states—and amenable to correction by H_2_S donors—include (243, 295) aging, ischemia, cardiac hypertrophy, heart failure (HF), liver disease (cirrhosis, steatosis), hypertension, atherosclerosis, endothelial dysfunction, diabetic complications, preeclampsia, Alzheimer's disease (AD), and Huntington's disease (HD). Further, Szabo and Papapetropoulos rightly point out that not only H_2_S-poor diseases can be corrected by H_2_S supplementation, since “there are also several indications where endogenous H_2_S levels are not suppressed, and yet H_2_S donation may be beneficial or warranted.” Additional evidence, obtained mainly after the list just cited was compiled, supports considering therapeutic application of H_2_S prodrugs in cancer (148), psoriasis (12), multiple viral infections (24, 25), colitis (180), autoimmune pathologies (42), systemic sclerosis (2), multiple sclerosis (245), Parkinson's disease (PD) (111), intracerebral hemorrhage (288), Duchenne muscular dystrophy/cardiomyopathy (44), allergic diseases (173), fibrotic disorders (229), osteoarthritis (164), osteoporosis (281), sarcopenia (32), pulmonary hypertension (287), ocular hypertension (225), kidney diseases (15), hearing loss (152), lens opacification (190), testicular dysfunction (262), male subfertility (178), erectile dysfunction (64), and periodontitis (89) as well.

**FIG. 2.**
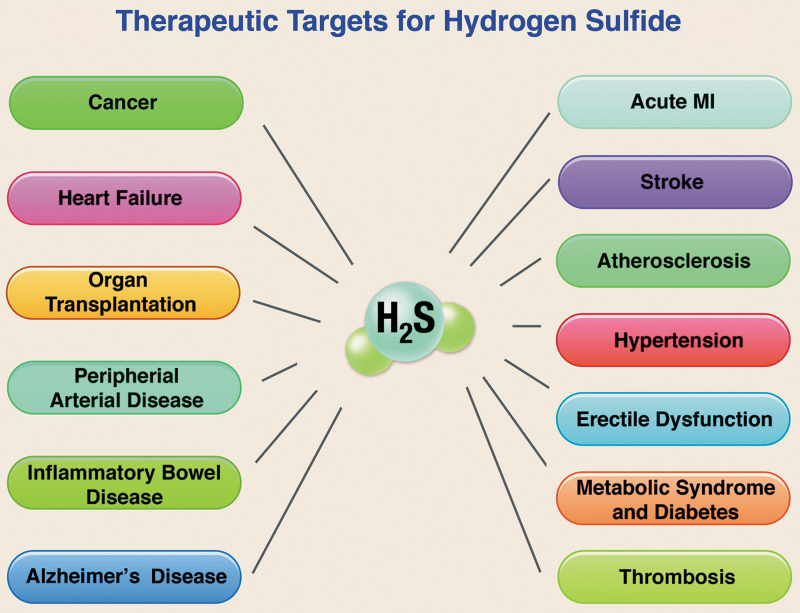
**Therapeutic targets for H_2_S.** H_2_S, hydrogen sulfide; MI, myocardial infarction. Reprinted with permission from Predmore *et al.* (202). Copyright 2012 Mary Ann Liebert, Inc., publishers.

At least in the cardiometabolic, cerebrovascular, and oncologic settings, current evidence supports the view that H_2_S-based treatments are often capable not only of slowing disease progression but also of leading to remission or long-term functional recovery and/or reversal of damage to tissues and organs (Fig. 2) (20, 109, 184, 224, 274).

### H_2_S is a pleiotropic multitargeted drug with immune-modulating and stem-cell-regulating properties

The pervasive and profound effects of H_2_S on so many diseases correlate with its pleiotropic actions on multiple molecular signaling pathways (81, 127, 128). It is now realized, on the basis of the large number of failed single-targeted drugs in clinical trials/development, that molecularly targeted therapies are far from being ideally suited for effectively treating highly complex disease states, such as cancer, human immunodeficiency virus–acquired immunodeficiency syndrome (HIV-AIDS), and diabetes, which require modifying integrated biologic responses rather than targeting single pathways.

The pleiotropic biological profile of H_2_S, which includes potent antioxidant, antiapoptotic, anti-inflammatory, vasoactive, and cytoprotective effects on normal (nontransformed) cells, can be harnessed to successfully treat such complex pathologic conditions. For accounts of this new paradigm, please see Sestito *et al.* (216) and Katselou *et al.* (122).

Although H_2_S is metabolized by normal (*i.e.*, nonmalignant) cells to generate benign cytoprotective chemical species (see Dynamic H_2_S regulation and therapeutic potential of sulfane—sulfur prodrugs section), it exerts marked proapoptotic effects on cancer cells through excessive reactive oxygen species (ROS) fluxes and intramolecular hyperacidification that correlate with a metabolic shift toward glycolysis (149, 248, 272). Moreover, its anticancer effects seem to be especially pronounced against progenitor cancer cells and against metastasis (148), so it is possible to envision the development of a truly disease-modifying H_2_S prodrug that is effective against many types of malignancies. The said drug should be capable of exerting on normal cells the same cytoprotective effects related to consumption of natural H_2_S precursors such as garlic or broccoli (26), bathing in H_2_S-rich springs, or drinking sulfurous water (26, 78, 250).

Both directly and indirectly—through the H_2_S-cysteine-glutathione connection (see The H_2_S-cysteine-glutathione connection section)—H_2_S regulates the homeostasis of the cellular immune system (169, 175). In addition, H_2_S is also able to improve progenitor-cell proliferation, viability, and therapeutic potential (1, 56, 97, 156, 283).

### H_2_S prodrugs as “medical foods”

Compositions of matter intended for the specific dietary management of a disease or condition, and orally or enterally administered under the supervision of a physician are eligible in several industrialized countries—including Japan—to receive marketing authorization as medical foods (United States), dietary foods for special medical purposes (European Union), foods for special dietary use (Canada), or foods for special medical purpose (China, Australia). If restoration of normal levels of H_2_S in patients of a given disease may be accomplished by treatment with an orally or enterally active H_2_S prodrug, it should be possible to reach the market much more swiftly and less costly through this channel.

### H_2_S prodrugs *versus* NO prodrugs

Sparatore *et al.*, after working for many years with both NO- and H_2_S-releasing derivatives of established drugs, have stated that “In the past, we worked for ∼15 years with NO donors, with hundreds of publications and patents, and we can say that H_2_S and the H_2_S-releasing drug hybrids are something beyond, and represent a significant technology advancement from, NO-donating hybrids. NO hybrids can sometimes be very toxic (causing genotoxicity) and poorly active (inducing tolerance) in redox imbalance conditions where NO is known to react with superoxide anion, leading to formation of peroxynitrite, a highly toxic molecular species. The clinical relevance of this characteristic is given by the evidence that tolerance/tachyphylaxis reactions by nitrates are one of the major problems associated with the long-term use of organic nitrates, whereas nitrites are known genotoxic agents in humans. In the case of H_2_S the situation is different because the superoxide anion is inactivated by H_2_S itself thanks to GSH formation, and no genotoxicity or tolerance occurs” (231).

### Dynamic H_2_S regulation and therapeutic potential of sulfane–sulfur prodrugs

Biological H_2_S levels are dynamically regulated (243). H_2_S may be oxidized (27, 168, 177) and readily detoxified by mammals: As soon as it comes into contact with blood or tissue, it is mostly oxidized into many highly biocompatible sulfane–sulfur bearing metabolites such as thiosulfate, hydropersulfides, and polysulfanes, from which H_2_S is then regenerated as needed and where needed through redox reactions. It is highly likely that the remarkable capacity of a mammal's blood to quickly detoxify large doses of HS⁻ (68, 257, 267) is associated with the rapid hemoglobin-catalyzed autooxidation of HS⁻ to thiosulfate, HSxH, and albumin-SSH.

In view of what has been just stated, prodrugs of any sulfane–sulfur metabolite may also be developed and might prove to possess an even higher therapeutic index than H_2_S prodrugs. In fact, prodrugs of hydrogen persulfide (H_2_S_2_) and biocompatible hydropersulfides have been synthesized and studied. Moreover, it is now clear that precursors of H_2_S, such as thiosulfate or SO_2_ (129), may bear S atoms with any oxidation number between minus two and plus four, and that some constituents of the SS pool are in dynamic equilibrium (29, 145, 168, 177).

### Systemic effects of H_2_S prodrugs

H_2_S is readily and efficiently translocated in our bodies, not so much because it is a small molecule, but because it is converted into highly biocompatible circulating metabolites bearing SS, such as thiosulfate and S-sulfhydrated human serum albumin (HSA; *e.g.*, albumin hydropersulfide). HSA is primarily an extracellular protein (188, 251), but it may reach most cells, be taken up to a certain extent, and finally be catabolized by lysosomal degradation, thus contributing to the maintenance of intracellular GSH levels. Further, recent evidence indicates that: (i) HSA is a major pool of SS (104, 105, 218) whose main constituent is albumin hydropersulfide, (ii) HSA hydropersulfide is a potent ROS scavenger, and (iii) there is a positive correlation between the SS content of semen and plasma.

Since even conditions that apparently affect only a specific organ—such as infertility, brain cancer, or psoriasis—are, in fact, systemic or at least partly driven by systemic signaling processes (182), the efficient translocation of H_2_S constitutes a highly desirable feature of H_2_S-based therapies and contributes to their wide-ranging applicability.

Glutathione, the master antioxidant that may be enzymatically synthesized by using H_2_S as a building block (see The H_2_S-cysteine-glutathione connection section), may also be efficiently transported by red blood cells (RBCs) and widely distributed by the blood. Its extracellular antioxidant capacity, that is, its ability to reduce extracellular oxidants *via* transmembrane electron transport, makes the RBC a powerful oxidant scavenger not only in its local environment but also throughout all plasma-accessible parts of the body (125).

### Proof-of-concept-of-sorts

#### Zofenopril

Zofenopril is a sulfur-containing drug belonging to the angiotensin converting enzyme (ACE)-inhibitor class. Zofenopril is metabolized to S-zofenoprilat, which is an active ACE inhibitor; it has been in the market since 1999 and has proved its safety and effectiveness in the cardiovascular setting beyond reasonable doubt (36, 37, 40, 69). However, zofenopril exerts cardiovascular-protective effects that are independent of and go beyond ACE inhibition; such beneficial ACE-unrelated effects have been shown by Bucci *et al.* (40) to stem from H_2_S donation by S-zofenoprilat. Although serendipitous and indirect, this may be construed as proof-of-concept that the development of H_2_S prodrugs is a viable endeavor.

#### Clopidogrel

Quite recently, Zhu *et al.* identified the widely used antithrombotic drug clopidogrel as an H_2_S prodrug (293). The establishment of clopidogrel as a serendipitous H_2_S donor that has been widely used in the clinic for more than 20 years “stands in sharp contrast with all rationally designed H_2_S donors,” since—at best—they are still in clinical trials or awaiting approval.

Before the expiry of its patent, clopidogrel was the second best-selling medication worldwide; it is now on the World Health Organization's List of Essential Medicines, the most effective and safe medicines needed in a national health system. The clopidogrel saga demonstrates that serendipity may also enable the development of safe and effective H_2_S prodrugs, and that we might soon witness the approval of serendipitously discovered H_2_S prodrugs as useful as penicillin, meprobamate, or sildenafil.

In fact, the overwhelming majority of the legion of researchers actively involved in this field all over the world remain optimistic and would agree that “generally, the concept of therapeutic H_2_S donation is well justified, because there are many pathophysiological conditions where endogenous H_2_S levels are suppressed, and donation (*i.e.*, “replacement therapy”) makes pathophysiological and experimental therapeutic sense” (243).

## Prodrug-Based H_2_S Supplementation Is an Attractive Strategy for Prolonging Healthspan and Lifespan

### The H_2_S-cysteine-glutathione connection

In a review article published in 2012, Predmore *et al.* (202) hypothesized that the H_2_S-cysteine-GSH connection (*i.e.*, the widely observed H_2_S-stimulated increase in intracellular glutathione levels) “is strongly dependent on the fact that H_2_S and L-serine act as co-substrates of the enzyme cystathionine beta synthase (CBS) to yield L-cysteine” (41, 67, 98, 137, 197); if this hypothesis is correct, exogenous H_2_S is capable not only of acting as a powerful antioxidant (mainly indirectly—*via* Nrf2 activation) but also of being used as a building block for the synthesis of cysteine, cysteine hydropersulfide, cysteine trisulfide, taurine, hypotaurine, thiotaurine, glutathione, glutathione hydropersulfide, and glutathione trisulfide. In other words, exogenous H_2_S may be used by the organism—when the diet is low in sulfur (186)—to synthesize an entire array of sulfur-bearing molecules needed for cytoprotection against free radicals, oxidants, electrophiles, xenobiotics, and infectious agents (170), including viruses (24, 25).

Glutathione (GSH) is the most abundant low-molecular-weight (LMW) intracellular antioxidant: In fact, GSH is quantitatively the most important scavenger of free radicals (74). Adequate levels of this tripeptide are essential for protecting cells against oxidative stress and the resulting dysregulation of redox-sensitive signaling pathways, for detoxification of xenobiotics and endogenous toxins such as 4-hydroxynonenal (214), for protecting spermatozoa from ROS-induced damage during epididymal maturation and storage (50, 106, 162), for checking the progression of inflammatory and degenerative conditions (including cancer, PD, and diabetic neuropathy), for its use as a cofactor in the biosynthesis of many essential metabolites such as leukotrienes, and for maintaining optimum immune function (19, 169).

The role of GSH as a master antioxidant is not limited to the cytosol, since it is able to transfer reducing equivalents across the plasma membrane in some cells (125), including RBCs (58); nuclear GSH has a critical role in the synthesis of DNA, the protection of DNA from oxidative damage and ionizing radiation, and the regulation of cell cycle (169).

The GSH values are suboptimal in a large number of wasting diseases, in hepatitis B, cystic fibrosis, AIDS, trauma patients, sepsis, degenerative conditions, old age, and after administration of certain medications such as acetaminophen, corticosteroids, and catabolic agents in general. Improvement in all of these conditions by supplying GSH prodrugs (mainly N-acetylcysteine) has been amply documented (38, 70–73, 75, 84, 157, 169, 186, 246).

### The H_2_S-cysteine-functional protein connection

In the frequent cases of deficient sulfur dietary inputs (186), exogenous H_2_S may also contribute—by increasing cysteine availability—to fight syndromes caused by suboptimal biosynthesis of vitally important cysteine-rich proteins such as the so-called “cysteine-rich secretory proteins” (CRISPs). The CRISPs are found only in vertebrates within the male reproductive tract; they have been implicated in many aspects of spermatogenesis, as well as in the actual process of fertilization (135), and downregulation of CRISP-2 mRNA by a factor of 4.3 in asthenospermic patients has been reported (112). A deficit of dietary sulfur compromises the synthesis of cysteine-rich structural proteins to an even greater extent, as evidenced by the significantly faster growth of nails observed on oral administration of SG1002 to many human subjects (unpublished data).

### H_2_S prodrugs as sulfite/thiosulfate/sulfate precursors

Sulfite, thiosulfate, and inorganic sulfate are generated through H_2_S catabolism; thiosulfate performs a fundamental role in cytoprotection, whereas sulfite has been shown to protect neurons from oxidative stress (129). Inorganic sulfate is employed in enzyme-dependent detoxification and serves as a building block in the synthesis of important biomolecules such as vitamin D3 sulfate, dehydroepiandrosterone sulfate, and sulfated glycosaminoglycans.

### H_2_S/SS as master cytoprotectors

The effector of cytoprotection is usually an SS species or a biomolecule produced *via* SS-induced Nrf2 nuclear translocation. The SS pool constitutes a defensive system against electrophilic stress/carbonyl stress (132, 133, 220). Emerging evidence indicates that electrophilic species—such as 4-hydroxy-2-nonenal (4-HNE) or quinones—are detoxified by formation of adducts with SS species, and it suggests that detoxification of heavy metals is mediated by sulfur atom transfer from an SS species to a metal atom in the +1 or +2 oxidation state (3, 9).

### H_2_S positively impacts the hallmarks of aging

Direct interference of H_2_S on pathways related to aging was identified in all but one of the hallmarks of aging (195), and it is likely that H_2_S exerts antiaging effects by inhibiting formation of advanced glycation end-products (161) as well as by blocking activation of their receptors (292). In fact, H_2_S has been considered “the next potent preventive and therapeutic agent in ageing and age-associated diseases” (289), and Zivanovic *et al.* (295) have stated that “dietary or pharmacological interventions to increase persulfidation associate with increased longevity and improved capacity to cope with stress stimuli.”

### Aging as a cysteine deficiency syndrome

A deficit of sulfur in the diet is known to compromise GSH synthesis to a much greater extent than protein synthesis, with potentially devastating consequences for the immune system, the antioxidant defense system, and the detoxification system (169, 186). In fact, several studies by Lang *et al.* (143), Droge (70, 71), Droge *et al.* (73), Droge and Kinscherf (74), and Droge *et al.* (75) led these authors to conclude that aging may be conceptualized as a cysteine deficiency syndrome.

### Critical nature of total sulfur input

The importance of adequate dietary sulfur inputs cannot be overstated. Available evidence supports the hypothesis that the single most important factor positively influencing both healthspan and lifespan is total sulfur intake, including sulfur in water (*e.g.*, sulfates, colloidal elemental sulfur, H_2_S, *etc.*) and sulfur in foods/food additives (*e.g.*, proteins, garlic, onion, broccoli, anamu, sulfated polysaccharides from algae, sulfated glycosaminoglycans, inorganic sulfites and sulfates, *etc.*); this evidence stems mainly from demographics, epidemiology, biomedicine, biochemistry, geochemistry, and nutrition research. The following are some of the most relevant pieces of evidence.

#### Evidence from population studies

The geographic distribution of healthy longevous individuals is far from uniform, and so is the distribution of sulfur in the biosphere and hydrosphere. It turns out that healthy supercentenarians abound only in sulfur-rich regions of our planet: The existence of these so-called “blue zones” has been thoroughly documented in several population studies (66, 191, 208).

Only five “blue zones” are currently recognized: the islands of Ikaria and Sardinia (Mediterranean Sea), the Pacific archipelago of Okinawa (Japan), the Nicoya peninsula (Costa Rica), and the community of Loma Linda (California); two additional “blue zones” are now emerging: the village of Acciaroli on the coast of the Tyrrhenian Sea in southern Italy and the southernmost tip of Sweden (Scandinavian peninsula). Of note, three out of the seven “blue zones” are located in or close to Italy (Sardinia, Acciaroli, Ikaria), which is the country with native sulfur deposits so plentiful that more than 50 million tons have been mined just from the Sicily area, most of it before 1900, and these deposits could again become a highly productive zone.

#### Evidence from global health statistics

According to the *Bloomberg 2017 Global Health Index* of 163 countries, a baby born in Italy in 2017 can expect to live to be an octogenarian, but 2800 miles south in Sierra Leone the average newborn will die by 52.

Italy was ranked in 2017—by the same source—as the world's healthiest country: Italians are, healthwise, in much better shape than Americans, Canadians, and U.K. citizens, who suffer from higher blood pressure, higher cholesterol, and poorer mental health.

The eight healthiest countries in 2017 (in ascending order) were Sweden, Japan, Spain, Australia, Singapore, Switzerland, Iceland, and Italy. Japan, Australia, Singapore, Iceland, and Italy are volcanically active (and consequently sulfur-rich) countries or are located close to volcanic areas.

In Sweden, Japan, Spain, Australia, Singapore, Iceland, and Italy, a large share of the population inhabits coastal areas where sulfate-loaded aerosols from seawater are carried by the wind and/or sulfated polysaccharides from algae are deposited on the shores.

In 2017, sulfur-rich Greece was the 20th healthiest country, outranking the much wealthier United Kingdom (23rd) and United States (34th). The three healthiest Latin-American countries were Chile (29th), Cuba (31st), and Costa Rica (33rd); Chile, in South America, is a long and narrow strip of land next to the Pacific Ocean with 90 active volcanoes; Cuba is an island; and Costa Rica is a small country located on the Central American Isthmus: It borders the Caribbean Sea and the Pacific Ocean and has five active volcanoes. Sulfur-rich Chile, Cuba, and Costa Rica also outranked the much wealthier United States.

## Dibenzyl Disulfide and Dibenzyl Polysulfides

### Anamu-derived organic sulfur compounds are benzyl analogues of garlic-derived organic sulfur compounds and display qualitatively similar chemical and pharmacologic properties

Dibenzyl tetrasulfide (DBTTS), dibenzyl trisulfide (DBTS), and dibenzyl disulfide (DBDS) are bioactive organic sulfur compounds (OSCs) that may be isolated from extracts of roots and leaves of the wild perennial shrub *Petiveria alliacea L* (Fam. Phytolaccaceae), also known as “anamu,” “apacin,” and “Guinea hen weed”; it is indigenous to the Amazon Rainforest, but it may now be found throughout subtropical areas of the United States, Mexico, Central America, South America, the Caribbean, and Africa.

The epithet “alliacea” refers to the pungent garlicky smell that results from plant tissue disruption; in addition to DBTTS, DBTS, and DBDS, many other OSCs have been isolated from *P. alliacea* extracts, including saturated sulfur heterocycles, dipropyldisulfide, thiosulfinates, and sulfoxides such as S-benzylcysteine sulfoxide. It is highly likely that this sulfoxide serves as a precursor to the isolated thiosulfinates and organic polysulfides bearing benzyl moieties in enzyme-catalyzed reactions that ensue as soon as the plant tissues are disrupted by crushing (22, 254).

Given the high similarity of the electronic effects exerted by the allyl and benzyl groups (92), it seems highly likely that not only the biochemical origins of the OSCs in garlic and anamu should present analogies, but also their chemical reactivity and biological properties. In effect, when Kaschula *et al.* (120) comparatively studied the antiproliferative effects of ajoene (allyl-(S = O)-CH_2_-CH = CH-S-S-allyl) and 12 ajoene analogues on WHCO1 esophageal cancer cells, they found that the analogues obtained on substituting either or both allyl groups by benzyl moieties were considerably more active: This observation is in line with the well-known fact that nucleophilic substitution reactions on benzylic carbon atoms are usually faster than on allylic positions (234). In this respect, it is convenient to bear in mind that both allyl and benzyl halides are—on average in S_N_2 reactions—more reactive than ethyl halides by factors of 40 and 120, respectively (234).

Moreover, Bhattacherjee *et al.* (28) recently stated that bis(4-cyanobenzyl)disulfide exhibited the highest antiproliferative activity, among all the 4-substituted benzyl analogues of diallyl disulfide (DADS) studied, in assays using MCF-7 human breast cancer cell lines. These authors found that the half maximal inhibitory concentration (IC_50_) values (μ*M*) for bis(4-cyanobenzyl)disulfide, DADS, and DBDS were 3.66, 16.76, and 43.60, respectively; whereas toward the normal kidney epithelial cell line (NKE) the respective values were 65.71, 45.43, and 70.11. They further found that both DADS and bis(4-cyanobenzyl)disulfide were cytotoxic toward cancer cell lines HepG2 (liver), PC-3 (prostate), and HCT-116 (colon), with each compound displaying IC_50_ values that clustered around that for the MCF-7 cell line.

Nowadays, herbal medications derived from *P. alliacea* are marketed in Japan, Paraguay, and Cuba, and several studies have revealed the therapeutic potential of DBTS as an immunomodulatory, anti-inflammatory, analgesic, antiviral (hepatitis C, HIV), and antiproliferative agent. Moreover, Williams reported in 2010 (268) that DBTS administration to old mice caused a 52% increase in thymus weight after 3 weeks of receiving 11 mg of DBTS/kg/day, as well as overall health improvement as judged by muscle tone and hair appearance.

On the basis of these and other results, Williams believes that DBTS administration may: (i) reverse the process of thymic involution (which sets in at about 28 years of age in humans), thereby delaying the onset of aging-related diseases such as osteoarthritis and some forms of cancer, and (ii) have a role in the treatment of inflammatory aging diseases.

The effectiveness of DBTS as an anticancer agent has been thoroughly documented *in vitro* in human cell lines (SH-SY5Y neuroblastoma, 5637 primary bladder carcinoma, Mia Paca-2 pancreatic, MCF-7 and MDA-MB231 breast, IPC melanoma, A2780 and OVCAR4 ovarian, K-562 and Jurkat leukemia, HeLa adenocarcinoma, PC-3 and DU145 prostate, A549 small lung, H460 nonsmall lung, HL-60 promyelocytic, HT1080 fibrosarcoma, TE-671 sarcoma) and murine Mel-Rel melanoma (13, 280) and *in vivo* in leukemic cows (22) and in mice bearing transplanted sarcoma S180 tumors and Lewis lung tumors (280). A clinical trial (ClinicalTrials.gov Identifier: NCT04113096) aimed at treating four types of cancers is now underway.

Williams *et al.* discovered that DBTS and serum albumin interact to yield a complex that prevents protein denaturation and displays 2000-fold enhanced cytotoxicity (*i.e.*, antiproliferative activity): Specifically, the IC_50_ value found for SH-SY5Y neuroblastoma cells is about 3 ng/mL (209, 270, 271); DBTS also displays high affinity for erythrocytic membranes, dose dependently increasing their elasticity and relaxation time (194).

The toxicity of DBTS is rather low: The mouse single intravenous dose LD50 value is 259 mg/kg (280); at a concentration of 10 millimole/L, it did not have any effect on the sensitive process of protein biosynthesis in Starfish (*Asterina pectinifera*) embryos, it was found not to be toxic (at 8.9 μ*M*) over 7 days toward HOFA human fibroblasts, and doses of up to 34 mg/kg did not cause mortality in mice (271). However, Pluth *et al.* (35) found that DBTS and DBTTS, at 100 μ*M* or higher concentrations, demonstrated “considerable cytotoxicity” toward bEnd.3 murine epithelial cells; DBTTS appeared to be significantly more cytotoxic than DBTS. The same authors assessed the cytotoxicity of metabolic by-product benzyl mercaptan (benzyl thiol [BnSH]) using S-benzyl ethanethioate (BnSAc) as the source of BnSH to enhance cell uptake, and they found no significant cytotoxicity at concentrations as high as 400 μ*M*.

Regarding possible drug–drug interactions, it has been found (181) that DBTS potently inhibits the activity of the cytochrome P450 enzymes. Therefore, adverse interactions may ensue on coadministration of DBTS and a drug that is eliminated *via* cytochrome P450-mediated oxidation.

### Is the anticancer action of dibenzyl polysulfides mediated by ROS or H_2_S?

At ambient temperature in the presence of cysteine (500 μ*M*, 20 equivalents), the H_2_S-releasing efficiency of DBTS after 90 min was 34%, and that of DBTTS 35%; under comparable conditions in the presence of GSH, the respective H_2_S-releasing efficiencies were 17% and 32% (35). However, evidence gathered from 2001 onward strongly suggests that most antiproliferative effects of DBTS, DBTTS, and garlic-derived disulfides and polysulfides are not mediated by H_2_S and are only modulated by ROS (13, 14, 94, 95, 107, 118–121, 124, 151, 209, 226, 227, 254, 276, 277, 279, 282).

Excessive ROS fluxes have often been hypothesized to be the root cause of cancer cell apoptosis (100, 203), and intracellular autooxidation of DBTS-derived (by reaction with GSH) benzyl hydropersulfide, undoubtedly, contributes to superoxide radical anion generation. In fact, Chatterji *et al.* (52) have shown that benzyl hydropersulfide readily autooxidizes to produce ROS under physiologically relevant conditions.

Let us now examine the evidence supporting selective protein S-thiolation-induced apoptosis/antimetastatic action as the central mode of anticancer action of garlic- and anamu-derived disulfides and polysulfides.

Several researchers have shown that treatment (*in vitro* or in transformed cell cultures) with low micromolar DBTS, bis(4-fluorobenzyl)trisulfide, and other DBTS derivatives, DBTTS, diallyl tetrasulfide (DATTS), diallyl trisulfide (DATS), DADS, S-allylmercaptocysteine, and ajoene or its analogues causes site-selective S-thiolation of beta-tubulin and disassembly of microtubules with ensuing cell cycle arrest in metaphase and apoptosis.

Ajoene also interferes with protein folding in the ER of human MDA-MB-231 breast and WHC01 esophageal cancer cells (121), arresting their growth and potentially causing apoptosis; this effect was shown to be mediated by S-thiolation of multiple protein targets, which interfered with protein folding, led to accumulation of misfolded protein aggregates, activated the unfolded protein response generating ER stress, and, eventually, resulted in apoptosis. In addition (121), ajoene S-thiolates vimentin at Cys328, disrupting the vimentin network, and hence exerts antimetastatic activity in human HeLa and MDA-MB-231 cancer cells.

An *et al.* (13) used a real-time cell electronic sensing system in cytotoxic screening assays of DBTS and several derivatives, including bis(4-fluorobenzyl)trisulfide: These compounds elicited the same kinetic response pattern as paclitaxel, an anticancer agent known to act by perturbing the tubulin–microtubule dynamic equilibrium. The same authors have provided additional evidence—stemming from experiments involving microscopic observation of purified tubulin—that DBTS disrupts the microtubule network.

It is possible to revert the effect of DBTS on cancer cell cycle and prevent apoptosis by adding thiols such as GSH or 2-mercaptoethanol (ME) to the culture medium (94); at the molecular level, this reversal is consistent with chemical reduction by thiols of the thiolated protein:

Pr-S-S-Bn+RSH→Pr-SH+BnSR

Kelkel *et al.* (124) studied DATTS-induced apoptosis of human histiocytic lymphoma U937 cells; they showed that DATTS effect was independent of ROS generation, that DATTS-induced tubulin depolymerization prevents formation of normal microtubules and leads to G2/M cell cycle arrest, that c-jun N-terminal kinase—which is activated early by DATTS—mediated phosphorylation, and that proteolysis of the antiapoptotic protein Bcl2 directly connects microtubule perturbation to apoptosis induction.

### Structure–activity relationships

A significant linear correlation between cytotoxicity toward WHCO1 esophageal cancer cells (Log IC_50_) and pKa of the leaving group (LG) of 12 ring-substituted disulfides and thiosulfonates of respective general formulas R-S-S-CH_2_CH_2_CH_3_ (LG = RS⁻) and R-S-SO_2_-C_6_H_4_-*p*-CH_3_ (LG = *p*-toluenesulfinate) was reported in 2016 by the Hunter-Kaschula group based at The University of Cape Town (227), lending support to a mechanism dependent on thermodynamically driven protein S-thiolation, with the thiolate leaving group stability paralleling the leaving group ability and cytotoxicity; these authors also obtained evidence pointing to a secondary role for ROS.

The finding that in an extraordinarily complex multifactorial multistep process, culminating with cancer cell death, it is possible to correlate the outcome with only one factor affecting only one step (a chemical reaction) is truly remarkable and attests to the power and usefulness of quantitative structure activity relationships. In this instance, to a first approximation the following steps may be considered: (i) transport of a molecule of the active compound to a cell, (ii) transport across the cell membrane and into the cytoplasm, (iii) transport through the cytoplasm from the endofacial side of the cell membrane to an intracellular protein, (iv) S-thiolation of a protein's free cysteine residue, and (v) apoptosis induction by the S-thiolated proteins.

Fortunately, it is often the case that one of the biological process steps (the slowest step) determines the overall rate, and then the outcome may be correlated with the parameter (or parameters) affecting only the rate-determining step (RDS). In some instances, it is possible to quantitatively correlate biological outcomes with only one parameter: If the RDS is a chemical reaction, this parameter is often pKa, a Hammett electronic substituent constant, or a Taft steric substituent constant (92). In the following paragraphs, we will describe a particularly interesting and pertinent linear correlation between the cytotoxicities (Log IC_50_) of a series of symmetric para-substituted DBDSs and Hammett's sigma substituent constants; the existence of this correlation was overlooked by the authors of the paper from which the data were taken (28).

Bhattacherjee *et al.* (28) studied the cytotoxicities (toward human MCF-7 breast cancer cells) of DBDS and of four symmetric para-substituted derivatives bearing fluoro, methoxy, cyano, and nitro substituent groups in both para positions; they found that the difluoro derivative and DBDS exhibited very similar cytotoxicities, that a decrease in potency accompanied the incorporation of nitro and methoxy groups, and that replacement of the para-H atoms by cyano groups led to a dramatic enhancement of cytotoxic activity.

The results just cited are very hard to rationalize, because the strong electron withdrawing groups cyano and nitro were reported to exert opposite effects on cytotoxicity; this consideration led us to the assumption that the nitro groups were undergoing fast enzymatic reduction to electron-releasing hydroxylamino groups (-NH-OH), catalyzed by the nitroreductases expressed by MCF-7 cells (160, 261, 290). When this assumption is made, a good (*r*^2^ = 0.8127) linear correlation between Log IC_50_ and Hammett's sigma substituent constant is obtained; if the point corresponding to the nitro substituent is omitted, an excellent correlation (*r*^2^ = 0.9847) ensues. It is important to point out that such a correlation between cytotoxicity and sigma values implies that cytotoxicity correlates with pKa of the thiolate leaving group as well, and this corollary fits well with the results reported by the Hunter-Kaschula lab and those just presented.

In contrast with the marked influence of structure on cytotoxicity, revealed by the earlier studies on disulfides, An *et al.* (13) found a very limited effect of most substituent groups on the cytotoxicity of DBTS derivatives toward A2780 ovarian, OVCAR4 ovarian, HT1080 fibrosarcoma, H460 nonsmall cell lung, MCF7 breast, M231 breast, HeLa adenocarcinoma, and Jurkat leukemia cells; however, bis (*p*-tert-butyl) benzyl trisulfide and bis(2,4,6-trimethyl)benzyl trisulfide were inactive, as well as bis(heteroaryl)trisulfides.

It seems that in these series of the trisulfides leaving group ability is uniformly good, but lipophilicity (in bis(*p-tert*-butyl) and bis(2,4,6-trimethyl)-benzyltrisulfides) and/or steric hindrance to thiolate attack has become excessive. This explanation is appealing, because the leaving group is now a hydropersulfide anion; the explanation offered by the authors, however, is based on differences in molecular flexibility. Importantly, these compounds possess very low cytotoxicities against HepG2 cells, and the more active, consequently, have the potential to be developed into antitumor agents.

Although—as already stated—the primary mode of anticancer action of DBTS seems to be inhibition of the tubulin–microtubule dynamic equilibrium, other contributing factors are excessive ROS generation and concomitant GSH oxidation to glutathione disulfide (GSSG) (203). Finally, we must acknowledge that other biological effects of DBTS are quite likely mediated by H_2_S, such as its immunomodulatory and thymic-activating effects (209, 269).

## DADS and Diallyl Polysulfides

### As H_2_S donors

Garlic-derived OSCs include—among others—DATTS, DATS, DADS, and diallyl sulfide (DAS); available evidence indicates that only DATS and DATTS are efficient H_2_S donors under physiologically relevant conditions.

Benavides *et al.* (26) were the first to report that DATS readily released H_2_S on nonenzymatic reduction by GSH in an aqueous milieu and to postulate that this gasotransmitter mediates the vasoactivity of garlic; they also showed that—in RBCs—glucose is needed for GSH recycling and proposed a model of cellular H_2_S production from garlic-derived organic polysulfides as well as of H_2_S function in the cardiovascular system. According to this model, diallyl polysulfide molecules with more than two sulfur atoms may react with exofacial RBC membrane protein thiol groups (Allyl-SSS-Allyl + 2 Pr-SH → H_2_S + 2 Pr-SS-Allyl) or cross the cell membrane to react with GSH (Allyl-SSS-Allyl + 2GSH → H_2_S + 2 GS-SAllyl); GSH may participate in transmembrane electron transfer to reduce exofacial disulfide bonds.

The mechanism of the reaction between DATS and GSH has been examined in detail (43, 153): The first step is nucleophilic attack of GS⁻ on one of the two alpha-sulfur atoms, so as to displace the allyl hydroperthiolate anion; please note that attack on the central (beta) sulfur would lead to displacement of a poorer thiolate leaving group. Although the reaction of DATTS with thiols has not been scrutinized in detail, it may be predicted to be intrinsically faster and to eventually lead to the release of twice as much H_2_S per mole.

DADS and DATS are the most widely used and studied H_2_S donors, usually in the context of cancer prevention and treatment (100, 142, 199, 203, 276, 284); their low toxicity, multiple targets (tubulin, ER, histone deacetylases, endothelial–mesenchymal transition-related proteins, angiogenesis, metastasis, cancer cell stemness), and broad effectiveness against cardiometabolic dysfunction and a large number of cancer types in different stages have been demonstrated *in vitro* and in animal models and xenografts. However, it seems that H_2_S does not trigger cancer cell apoptosis in these cases; substantial experimental evidence supporting selective protein S-thiolation as an apoptosis trigger was presented in the preceding section.

Evidence that H_2_S does mediate other biological effects of DATS has been obtained in the settings of modulation of inflammatory stimuli, cardiovascular protection, hepatoprotection, and neuroprotection (26, 53, 65, 91, 179, 201, 249, 285).

Overall, it is clear that DATTS and DATS do not lend themselves to be used as tools for the study of H_2_S interaction with biological systems on account of the fact that they generate byproducts—both inert and bioactive—along with H_2_S and of the multiplicity of non-H_2_S-mediated cellular effects that they have been shown to exert. It may be said that—to some extent—H_2_S is just a byproduct of their biological actions. This is regrettable, because they are efficient donors that are able to release H_2_S under physiologically relevant conditions at rates that may be modulated by the nature and concentration of the thiolate reaction partner and to some degree by pH. In fact, DATS is a faster H_2_S donor than DBTS and DBTTS (35) in aqueous systems in the presence of GSH, and it yields about the same amount of H_2_S as DBTTS after 110 min: Bolton *et al.* attributed this to the higher stability of Bn-SSH in comparison to Allyl-SSH.

### As pharmacologic agents

Intriguingly, DAS has also been shown to display broad-spectrum anticancer activity in spite of its inability to donate H_2_S and to trigger protein S-thiolation; in this instance, the trigger is likely oxidative activation by ROS (62, 100) to a diallylsulfoxide (allyl-(S = O)-allyl) and/or diallylsulfone (allyl-(SO_2_)-allyl) (232). These chemotypes are susceptible to nucleophilic attack by biological thiols on allylic carbon, with protein S-allylation as the outcome.

Alternately, diallylsulfone may isomerize to the resonance-stabilized allyl-(1-propenyl)sulfone (allyl-SO_2_-CH = CH-CH_3_), an avid electrophile susceptible to Michael addition of thiols.

As human populations have been exposed to garlic-derived OSCs throughout recorded history, they do not elicit the toxicity concerns that accrue to most synthetic donors. DATS is commercially available with a purity as high as 98%, and its administration (orally and by parenteral routes) to experimental animals has been shown to safely increase the blood concentration of both H_2_S and SS to pharmacologically significant levels and to exert cytoprotective effects on normal cells. Moreover, the pharmacokinetic parameters of DATS in rats have been determined (237) and the metabolism of several OSCs (including DADS and DATS) has been studied, as discussed next. Therefore, it seems to us—and to many authors of hundreds of studies—that DATS is an excellent candidate for clinical translation.

According to Allah *et al.* (11), the development of DATS and DATTS has been hampered by “their low aqueous solubility, a certain chemical instability at room temperature, and above all, their pungent smell”: Many authors have expressed similar opinions, especially with respect to poor solubility in aqueous media. However, medicinal chemists have developed several powerful nanotechnological methods for dramatically increasing the apparent water solubility and bioavailability of hydrophobic drugs and are actively engaged in applying them to DATS (238, 239, 263, 264, 291). These methods rely on the concept of “supramolecular tuning” of drug properties, that is, instead of modifying a drug's molecular structure by “scaffold tuning,” they aim at incorporating the drug molecules into nanoscopic or microscopic entities that are usually amenable to formulation as aqueous systems. Regarding DATS chemical stability at room temperature, there are many drugs in the market that display some instability at room temperature and must be kept under refrigeration, but that hardly detracts from their usefulness.

As to the pungent smell of DATS, it is true that some of its metabolites (prominently allylmethylthioether) are ill-smelling and have a lingering presence in human breath and body odor, but we very much doubt that a cancer patient will object to a life-saving treatment on that basis, especially if such treatment does NOT require harsh chemotherapeutic agents but rather involves an immunity-optimizing drug with a high therapeutic index and mild GI side effects that will protect his/her vital organs from damage by other medications.

It has been established that the H_2_S-releasing step—that is, nucleophilic attack of GS⁻ on the inner (alpha) sulfur of allylhydroperthiol—also generates S-allylmercaptoglutathione (allylS-SG), a reactive disulfide that is eventually reduced to allylmercaptan (allyl thiol) in the GSH-rich intracellular milieu (176); as pointed out earlier, some allylmercaptan is excreted through the lungs and skin after undergoing enzyme-catalyzed methylation to allylmethylthioether. This metabolite of DATS (also known as allylmethylsulfide) is bioactive and has been shown to exert anticancer/antimetastatic effects via inhibition of histone deacetylases and attenuation of hypoxia (176, 266).

Further, emerging evidence indicates that long-lived metabolites derived from allylmethylthioether are also bioactive, mitigating hyperglycemia/liver damage and attenuating cardiac hypertrophy/remodeling in animal models (126, 235, 236); other authors recently found that the thioether is oxidized to allylmethylsulfoxide and allylmethylsulfone by liver microsomes (159). We propose that allylmethylsulfone readily isomerizes to highly electrophilic (1-propenyl)methylsulfone, which adds glutathione or cysteine; the adduct may then be excreted as such or as the corresponding mercapturic acid.

### As lead molecules

Later, we will address the assumption that other organic polysulfides with longer sulfur chains and/or tunable scaffolds will surely turn out to be as safe as DATS but better H_2_S donors, more bioactive, and less open to stench-derived objections, provided we do not deviate from the tenets of rational drug development.

#### Benzyl moiety-bearing lead molecules

In a 2015 article, Allah *et al.* (11) described the synthesis of two novel and rationally designed organic polysulfides with anticancer effects and “improved physico-chemical properties”; one of them (allylbenzyltetrasulfide) is a “hybrid” of DATTS and DBTTS, and the other is allyl(2-ethoxyethyl)trisulfide. The authors state that “the new odorless tri- and tetrasulfanes exhibit a similar activity compared to their natural counterparts, yet are easier to handle and also deprived of the offensive odor which so far has prevented most practical applications of such polysulfanes, at least in the context of medicine.”

However, the results obtained by these authors are disappointing: The new OSCs are viscous oils, and water-insoluble allylbenzyltetrasulfide displayed higher proapoptotic activity toward cancer cells than the water-soluble trisulfane but comparable to that of “its natural counterpart.” It seems that being nearly odorless is the only advantage of the tetrasulfide, since it is not obvious why the authors claim that they are easier to handle, but this putative advantage is inconsequential because ingestion or injection will necessarily lead to the same ill-smelling metabolites of DATTS or DATS (vide supra) plus additional foul-smelling metabolites such as benzyl mercaptan and methylbenzylthioether.

Another putative advantage of organic polysulfides bearing a benzyl group is—according to Allah *et al.*—that “many derivatives become feasible,” presumably because the same synthetic route that led to allylbenzyltetrasulfide may then be used to synthesize different ring-substituted derivatives, allowing experimenters to optimize the pharmacological profile through scaffold tuning. Once again, the available experimental evidence (vide infra) does not support this assumption.

As previously mentioned, An *et al.* (13) reported a very limited electronic effect of substituent groups on the cytotoxicity of DBTS ring-substituted derivatives against eight lines of human cancer cells: We hypothesize that in this series of the DBTSs leaving group (*e.g.*, hydropersulfide anion), ability is uniformly good. In other words, the leaving group-stabilizing or -destabilizing capacity of the ring substituents is rather small on account of both a saturation effect and the considerable distance between the substituent and the reacting trisulfide sulfur atom. Smith *et al.* (227) found that the cytotoxicity of a series of benzyl thiosulfonates (R-S-SO_2_-*p*-tolyl) was essentially the same for R = benzyl, R = *p*-fluorobenzyl, and R = *p*-methoxybenzyl, a result that may also be explained by invoking a reactivity-leveling effect related to high leaving group (*p*-tolylsulfinate) ability and poor transmission of electronic effects through six atoms.

In their study of competitive oxidation by *m*-chloroperbenzoic acid of ring-substituted DBTSs, Stensaas *et al.* (232) found that DBTS and bis(4-chlorobenzyl)trisulfide were oxidized at the same rate to the corresponding trisulfane 1-oxides (Aryl-CH_2_-(SO)-SS-CH_2_-Aryl); they conclude that “the terminal sulfurs in substituted DBTSs are not susceptible to electronic effects due to substituents on the phenyl groups.”

#### Diphenyl tetrasulfide as potential lead molecule

Experimental findings from Michael Pluth's laboratory (48) reinforce the notion that effective tuning of H_2_S release and biological activity of H_2_S donors is difficult to achieve by conventional means. Thus, they measured the initial rate constants for H_2_S release from six para-disubstituted diphenyltetrasulfides (Ar-S-S-S-S-Ar) reacting with dilute aqueous GSH present in considerable excess. In spite of the fact that the substituent's electronic effects differed widely, it was found that most of the observed initial rates differed only by factors of less than 7, and that para-substitution consistently led to substantially diminished rates.

It is noteworthy that the rate of reaction of the parent compound (diphenyltetrasulfide) was found to be 25 times greater than that of DATS, that this is expected on substitution of the electron-withdrawing phenyl group for the electron-donating allyl group (92) and that this observation also fits well with the fact that 7-methyl-1,2,3,4,5-benzopentathiepin is about 30 times more reactive than DBTS toward GSH (51).

#### Organic tetrasulfides and higher polysulfides as potential lead molecules

Another common assumption (35, 49) is that—ceteris paribus—longer sulfur chains in organic polysulfides lead to both greater bioactivity and higher reactivity toward thiolate anions, as well as to higher H_2_S yields, but a saturation effect seems to operate (35, 286), and stability is expected to be increasingly compromised as the number of catenated sulfur atoms grows (16, 62, 118). In addition, the therapeutic index of DBTS was found to be greater than that of DBTTS (35). Moreover, the molecular weight of DBTTS already exceeds the limit imposed by the “rule of three” (RO3) (see Druglikeness, RO5, RO3, Ghose filter, Veber's rule section), and its lipophilicity is too high from the perspective of Lipinski's rule of five (RO5) (63).

It is worth noting: (i) that the generalization regarding dependence of bioactivity on the number of tethering sulfur atoms is—to the best of our knowledge—based on the results of only a few studies focused on dimethyl(poly)sulfides (H_3_C-Sx-CH_3_), diallyl(poly)sulfides (allyl-Sx-allyl), and dibenzyl(polysulfides (Bn-Sx-Bn), in which the number (x) of catenated sulfur atoms was varied between one and four, and (ii) that only trisulfides were claimed by Xu, An, and Wang in U.S. Patent 8,334,316 (assigned to ACEA Therapeutics, Inc.), despite the fact that more than 150 novel organic polysulfides were synthesized and assayed; their molecules comprise phenyl, substituted phenyl, benzyl, substituted benzyl, phenethyl, and 3-phenyl-2-propenyl moieties, as well as heterocyclic moieties.

Remarkably, Xu *et al.* (280) found that DBTS—which they used as a reference standard in their anticancer assays—consistently displayed the highest anticancer activity. As already mentioned, toxicity against normal cells may increase on increasing the number of tethering sulfur atoms, as shown by Pluth *et al.* (35) for DBTS and DBTTS, and by Schneider *et al.* (213) for DAS, DADS, and DATTS.

#### DATS as lead molecule

Overall, it seems that the DATS molecule possesses just the right combination of sulfur content, electrophilicity, steric accessibility, and lipophilicity/cell membrane permeability; that its structure endows it with very low toxicity against normal cells and high bioavailability; that at least five of its metabolites (H_2_S, allylmercaptan, allylmethylsulfide, alylmethylsulfoxide, and allylmethylsulfone) are bioactive, with at least three being long-lived as well; and that this constellation of properties will prove to be very hard to emulate and even harder to improve on.

In fact, Iitsuka *et al.* (103) were unable to obtain an LMW organic trisulfide that was more bioactive than DATS through “scaffold tuning” in the setting of cancer cell growth inhibition (Table 1). These authors synthesized five saturated symmetric trisulfides, three unsaturated symmetric trisulfides, and allylmethyltrisulfide. They found that three compounds presented the lowest IC_50_ values, namely DATS, di(3-butenyl), and di(n-propyl) trisulfides, and were able to correlate inhibitory activity with lipophilicity (clogP value).

**Table 1.** cLogP and IC_50_ Values for HT-29 Colon Cancer Cell Growth Inhibition by Saturated, Unsaturated, and Mixed Organic Trisulfides

Compound name	cLogP^a^	IC_50_ (μ*M*)^b^
Dimethyltrisulfide	1.84	24.96
Diethyltrisulfide	2.59	16.83
Di(n-propyl)trisulfide	3.59	7.18
Di(n-butyl)trisulfide	4.71	17.48
Di(n-pentyl)trisulfide	5.72	33.32
Dially ltrisulfide	3.13	7.25
Di(3-butenyl)trisulfide	3.67	7.36
Di(4-pentenyl)trisulfide	4.68	19.72
Allylmethyltrisulfide	2.48	20.49

Adapted from Iitsuka *et al.* (103).

^a^ Values obtained by using the Molinspiration LogP Online Calculator.

^b^ Values taken from Iitsuka *et al.* (103).

Light gray = saturated trisulfides.

White = unsaturated trisulfides.

Dark gray = mixed trisulfides.

IC_50_, half maximal inhibitory concentration.

**FIG. 3.**
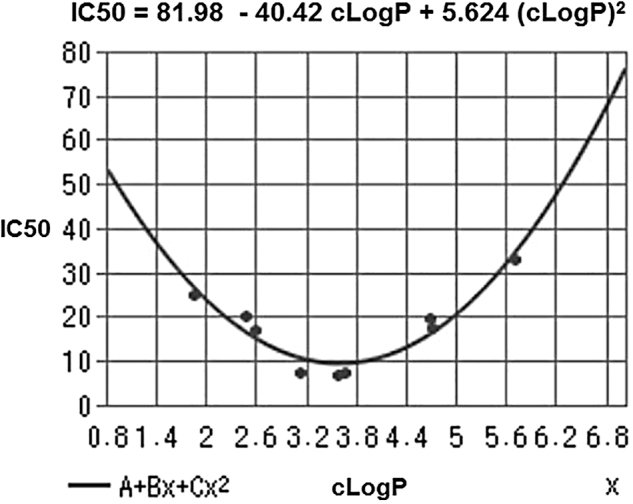
**cLogP and IC_50_ values for HT-29 colon cancer cell growth inhibition by saturated, unsaturated, and mixed organic trisulfides.** IC_50_, half maximal inhibitory concentration. Adapted from Iitsuka *et al.* (103).

The mathematical function relating IC_50_ to clogP was parabolic (Fig. 3). Importantly, the cLogP values of the three highly cytotoxic trisulfides lie between 3.13 and 3.67. Oosthuizen *et al.* (189) reported a similar outcome, as DATS showed the highest antimycobacterial activity when compared with DAS, DADS, and higher diallylpolysulfides.

It is likely that DATS (cLogP = 3.13) and DATTS (cLogP = 3.63) excel as lead candidates on account of both the electronic effect of the allyl group on reactivity and their optimal cLogP values. Lipophilicity affects receptor binding, plasma protein binding, metabolic drug stability, and drug distribution/excretion, and it is therefore a crucial physicochemical property of a drug molecule. In drug development, “lipophilicity is a pivotal and early indicator of the potential *in vivo* pharmacokinetic and dynamic behavior” (258).

Moreover, an adapted version of RO5 (see Druglikeness, RO5, RO3, Ghose filter, Veber's rule section) states that a LogP range of 2.0 to 3.5 is a fundamental predictive factor for blood–brain barrier penetrability *via* passive diffusion (265). In line with this modified RO5, the frequency distribution of cLogP values of more than 3000 drugs on the market resembles a gaussian curve and leads to the inference that drug-likness increases as the value of cLogP approaches 3.0 (204). On the other hand, although the lipophilicity of DBTS (cLogP = 5.03) and DBTTS (cLogP = 5.53) is suboptimal, emerging evidence (62, 120, 280) suggests that these molecules will prove to be as bioactive and useful as DATS and DATTS: We hypothesize that their suboptimal lipophilicity is compensated—or possibly overcompensated—by an enhanced reactivity since the benzyl moiety is more electron withdrawing than the allyl group (234).

However, we do not share the frequently held belief in the impossibility of subjecting DATS to a series of structural modifications to optimize therapeutic index/potency/efficacy through the establishment of a structure–activity relationship and using as guidance both the relationship mentioned earlier between bioactivity and LogP and the knowledge gathered on DATS metabolism.

## Heterocyclic Polysulfides

Varacin (an anticancer agent), lissoclinotoxin A, and lenthionine are examples of naturally occurring heterocyclic polysulfides that are capable of releasing H_2_S on reaction with biological thiols.


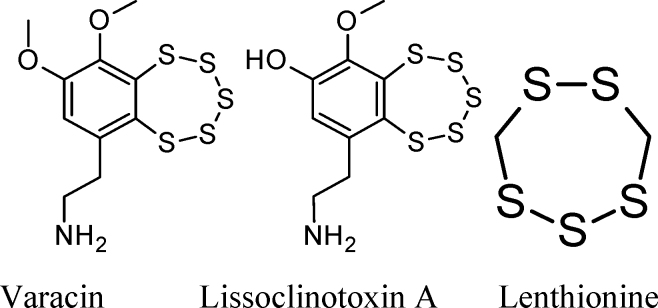


### Chemistry of a typical benzopentathiepin

Chatterji and Gates studied the reaction of GSH and ME with 7-methyl-1,2,3,4,5-benzopentathiepin, I (51):


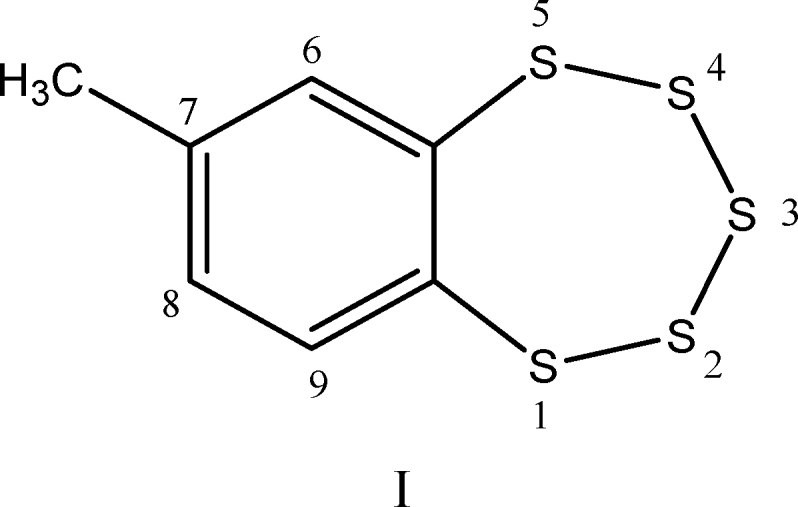


This simple synthetic compound contains the same benzo-annelated polysulfur ring system found in the bioactive natural products varacin and lissoclinotoxin A, as well as in synthetic H_2_S donors such as TC-2153 (see TC-2153 section).

These authors found that: (i) reaction of a thiol (GSH or ME) with compound I is quite rapid under physiologically relevant conditions. The half-life of I (6.25 μ*M*) in the presence of GSH (188 μ*M*) in buffered water–acetonitrile solution at 25°C is less than 1 min, which corresponds to an apparent second-order rate constant of about 60 *M*^−1^s^−1^. This reaction is significantly faster than the one between dibenzyltrisulfide and GSH under the same conditions (k_2_ = 1.77 ± 0.06 *M*^−1^s^−1^).

This result is consistent with computational studies on the attack of HS⁻ on the pentathiepin system, which point to a very low activation barrier (88), and with the results (49, 51) stemming from experimental determination of the reactivities of diphenyl tetrasulfide and DBTS toward aqueous GSH (see Diphenyl tetrasulfide as potential lead molecule section).

(ii) reaction of compound I (1 mole) with 100 mole of thiol (RSH) for 1 h yields ∼3 mole of H_2_S, 3.0 ± 0.25 mole disulfide (RSSR), and the aromatic dithiol 3,4-dimercaptotoluene (compound II):


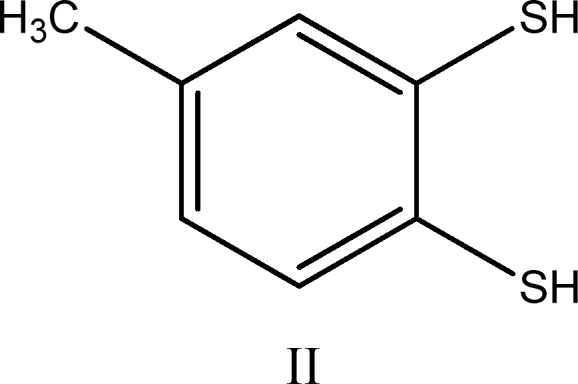


Compound II was isolated in 80% yield as its bis(methylthioether) derivative after workup of the reaction mixture with methyl iodide.

Unfortunately, Chatterji and Gates did not provide an explanation for the observed stoichiometry:


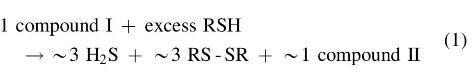


We believe that the observed stoichiometry corresponds to a weighted average of the stoichiometries accruing to eight or more simultaneous individual chemical reactions. It is highly likely that the first step in the mechanism of these processes is the nucleophilic attack of RS⁻ on a sulfur atom attached to the aromatic nucleus (*i.e.*, S1 or S5) or on a sulfur atom adjacent to it (*i.e.*, S2 or S4), with concomitant cleavage of the weaker S1–S2 or S4–S5 bonds and formation of either a thiolate (ArS⁻) or a hydropolysulfide (Ar-S-S-S-S⁻) relatively stable anion. In subsequent steps, the polysulfur chains are shortened with simultaneous generation of either HS⁻, a disulfide (RSSR), or a polysulfide (RS_x_R).

A detailed analysis of reaction pathways reveals that one should expect the formation of four main benzene derivatives: the dithiol (II), the bis-disulfide (III), and the two isomeric thiol-disulfides (IV and IV′).


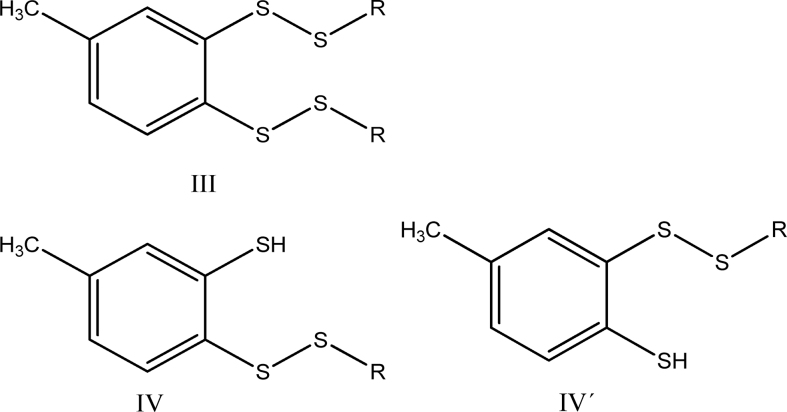


The reactions leading to II, III, IV, and IV′ possess the following stoichiometry:

(2) $$\begin{array}{cc} & 1compoundI+8RSH\to 1compoundII+4RSSR\\ & +3H_{2}S \end{array}$$

(3) $$\begin{array}{cc} & 1compoundI+6RSH\to 1compoundIII+2RSSR\\ & +3H_{2}S \end{array}$$

(4) $$\begin{array}{cc} & 1compoundI+7RSH\to 1compoundIV\left(orIV\prime \right)\\ & +3RSSR+3H_{2}S \end{array}$$

If reactions leading to compounds II and III have similar rates—which is a reasonable assumption on mechanistic grounds—the observed stoichiometry would be as follows:


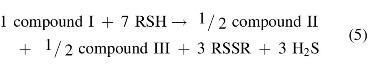


And if during workup—which involves treating with aqueous NaOH and heating at 50°C for 1 h—compound III is converted into II *via* nucleophilic cleavage of the sulfur–sulfur bonds:

(6)


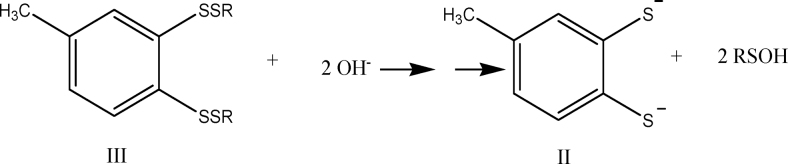


the observed stoichiometry would be:

(7) $$\begin{array}{cc} & 1compoundI+7RSH\to 1compoundII+3RSSR\\ & +3H_{2}S \end{array}$$

which is precisely the result reported by Chatterji and Gates. The same stoichiometry would be observed for reaction (4) if compounds IV and IV′ are also converted into compound II on workup.

The same researchers conclude that: (i) the pentathiepin ring system is likely to react rapidly with cellular thiols under physiological conditions, and (ii) the observed products are completely consistent with the previous proposal that thiol-triggered DNA cleavage by compound I proceeds *via* production of highly reducing polysulfide anion intermediates (RSSxS⁻) that convert molecular oxygen to the superoxide radical, thereby initiating the reaction cascade that ultimately yields DNA-cleaving radicals as shown in equations 8, 9, and 10:

(8) $${RSSxS}^{-}+O_{2}\to O_{2}{∙}^{-}+RSSSx∙$$

(9) $$2O_{2}{∙}^{-}+2H^{+}\to H_{2}O_{2}+O_{2}$$

(10) $$H_{2}O_{2}+M^{n+}\to HO∙+HO^{-}+M^{\left(n+1\right)+}$$

In equation 10, M^+^ represents a transition metal ion.

When 7-methylbenzopentathiepin reacts—at room temperature—with ME in chloroform containing a trace of triethylamine, the reaction mixture initially develops a bright yellow-orange color (indicative of polysulfide anion formation), which fades over the course of 15 min (51).

Chatterji and Gates point out that their findings raise the possibility that the anticancer effect of benzopentathiepins might be mediated not only by ROS but also by the H_2_S generated in the reaction with endogenous thiols (eqs. 2, 3, and 4) and/or by thiolation of cellular proteins.

### Thiozone: a benzopentathiepin-derived reactive sulfur species

In 2001, Greer (88) proposed—on the basis of a theoretical study—that the initially formed aromatic tetrasulfide anion collapses unimolecularly by S-S bond cleavage, generating thiozone (S_3_) and an aromatic thiolate anion. This intriguing hypothesis opened still another possibility, namely that S_3_ contributes to the anticancer action of benzopentathiepins. Two years later, Brzostowska and Greer (39) presented experimental evidence supporting pentathiepin desulfuration *via* S_3_ transfer and postulated that the amino group in varacin promotes its reaction with nucleophiles and concomitant S_3_ formation.

In 2010, Greer *et al.* (166) described the synthesis and *in vitro* anticancer properties of a number of highly cytotoxic hydrophilic PEGylated benzopentathiepins, and in 2016 the Greer lab reported (165) on the synthesis, characterization, and *in vitro* anticancer properties of a lipophilic ceramide analog. On both occasions, antiproliferative effects were studied only on human malignant cell lines; therefore, at this time it is extremely difficult to estimate the probability of successful clinical development of an anticancer drug based on a pentathiepin pharmacophore.

### TC-2153

#### Synthesis and pharmacologic development

8-(Trifluoromethyl)-1,2,3,4,5-benzopentathiepin-6-amine hydrochloride was first synthesized—circa 2009—at the Novosibirsk Institute of Organic Chemistry (Novosibirsk, Russia), is covered by patent RU0002672472 issued in 2018, and is being developed as an “antidepressant of new generation” by the research group of Alexander Kulikov (138, 139).

On the basis of preclinical studies in mice, Kulikov *et al.* were able to demonstrate that: (i) TC-2153 action is mediated by its effects on both the brain serotoninergic system and the brain-derived neurotrophic factor, which are known to be involved in the psychopathology of depression, and (ii) enteral (per os) and parenteral (intraperitoneal [ip]) routes of administration are essentially equivalent.

Five years ago, Lombroso and colleagues, at Yale University, identified 8-trifluoromethyl-1,2,3,4,5-benzopentathiepin-6-amine hydrochloride (TC-2153) as a potent inhibitor of striatal-enriched protein tyrosine phosphatase (STEP), an enzyme that is overactive in several neuropsychiatric, neurodegenerative, and aging-related cognitive disorders, including AD and HD (278).

Lombroso and his colleagues initially screened about 150,000 commercially available compounds, assessing their ability to inhibit STEP's activity, toxicity, and ability to cross the blood–brain barrier: Finally, eight good candidates emerged. Next, they synthesized the eight compounds “from scratch,” but it turned out that they possessed very little STEP inhibitory activity. They, therefore, considered the possibility that a contaminant present in the commercial lead compounds was inhibiting STEP activity, and discovered that, indeed, elemental alpha-sulfur was the active contaminant.

At this point, they felt that the unconventional molecular structure and properties of alpha-sulfur were definitely NOT those of a good “lead compound,” since its aqueous solubility is almost nil and its molecular structure does not enable further refinement through the preparation and evaluation of analogues that are potentially able to display higher aqueous solubility, binding affinity, and selectivity. Lombroso and colleagues then looked for molecules structurally related to cyclooctasulfur, and they “identified the benzopentathiepin core structure as the most promising for further investigation.” Eventually, they found that benzopentathiepin derivative TC-2153 had “reasonable aqueous solubility,” low acute toxicity (>1000 mg/kg), was able—on parenteral administration—to cross the blood–brain barrier of mice, and inhibited STEP almost as potently as S_8_.

These researchers employed the “triple transgenic” mouse model of AD, with mutations in genes known to cause this pathology (presenilin-1, amyloid precursor protein, and tau), to test the hypothesis that TC-2153 could reverse some of the cognitive deficits due to STEP overactivity: In comparison with vehicle, ip injection of TC-2153 significantly improved spatial working memory, novel object recognition, and reference memory.

#### Mechanism of STEP inhibition by TC-2153

Lombroso and colleagues found that inhibition could be reversed by incubation with thiols such as glutathione or dithiothreitol and proposed that it involved covalent modification of the active site cysteine (*e.g.*, Cys472) of the enzyme, stating that “our intact protein analyses (liquid chromatography-mass spectrometry) suggest a covalent adduct to STEP,” but they were unable to obtain the accurate mass of the adduct. In addition, they presented evidence (from liquid chromatography-tandem mass spectrometry examination of the peptides formed after in-gel tryptic digestion) of the “presence of a “*de novo*” trisulfide within the Cys465-Cys472 bridge, which was not observed for wild-type STEP alone. These authors conclude that “the active site cysteine is likely modified by TC-2153” and suggest that “following tryptic digestion a sulfur from the benzopentathiepin core is retained, giving rise to the trisulfide identified by mass spectrometry.”

We agree in general with their interpretation of these results, but have reservations regarding both the implication that a stable covalent adduct is initially formed—which, by definition, should comprise all atoms initially present in both the protein and inhibitor molecules—and the notion that this adduct is converted into the trisulfide on tryptic digestion. Rather, we believe that an enzyme trisulfide may be formed at an early stage, probably after the loss of a molecule of H_2_S from intramolecular disproportionation involving the initially formed Cys465-S-SH and Cys472-S-SH bishydropersulfide. This alternative interpretation is consistent with the similar effects of S_8_ and TC-2153 on STEP activity both *in vitro* and *in vivo*, as well as with the reactivity profile of S_8_, organic polysulfides, and hydropersulfides.

The work of Kulikov's group and the serendipitous discovery of Lombroso and his colleagues have had significant impact and might lead to the eventual development of a drug that is useful for treating depression, AD, HD, age-related cognitive impairment, schizophrenia, and drug abuse, but—for the time being—their pioneering work has led to a better understanding of how depression and cognitive dysfunction originate and how they may be fought. In addition, their work has decisively contributed toward opening new avenues to develop drugs targeting a category of enzymes that for a long time had been considered undruggable.

In addition, the findings that both TC-2153 and S_8_ are effective regardless of whether they are administered enterally or parenterally, and that their effect is mediated by a neuron-specific enzyme allow us to hypothesize that they may be prodrugs, with three or eight sulfur atoms in their molecules initially being converted into circulating H_2_S/SS, which is then transported into the brain. This is an attractive hypothesis, because S_8_ can be reduced to H_2_S by cells of many tissues and specifically by RBCs (26, 215), so it is highly unlikely that S_8_ molecules might survive unchanged during their long journey from the intestine to the brain. The benzopentathiepin polysulfur ring, as we have seen, is also susceptible to nucleophilic attack by biological thiols, including protein thiol moieties (55) with concomitant generation of H_2_S.

One further consideration is in order: It might eventually turn out that Lombroso and colleagues were too quick to dismiss S_8_ on the basis of uncritical adherence to an old paradigm, that is, of its very low water solubility and the impossibility of optimizing its properties by structural modification, because there are nowadays several ways to overcome such limitations. On the one hand, it is usually possible to dramatically increase the water solubility and to simultaneously improve the hydrophobic drug's pharmacokinetic and pharmacodynamic properties by “encapsulating” the drug in liposomes or in nanoscopic self-assembling micelles of amphiphilic polymeric “carriers” such as a poloxamer or poly(ethylene glycol)-block-polycaprolactone; on the other hand, the established drug development paradigm is hardly applicable to supramolecular entities, such as a drug-loaded nanoscopic micelle or an ion-doped drug crystal.

Finally, if a class Type II-Subtype A prodrug of a brain-penetrating drug is being designed, a medicinal chemist does not have to worry about the ability of the prodrug to cross the blood–brain barrier.

## SG1002: An Overview

SG1002 is a water-insoluble, alpha-sulfur rich (about 99% S_8_) microcrystalline material containing traces of ionic substances (sodium sulfate, sodium thiosulfate, and sodium polythionates) that strongly influence its physicochemical behavior. SG1002 is definitely not a simple mixture of alpha-sulfur and ionic materials and it is best conceptualized as supramolecularly modified alpha-sulfur or as alpha-sulfur doped (hydrophilized) with ionic materials to enhance its bioavailability.

The chemical synthesis (*via* comproportionation of sulfur atoms in the −2 and +4 oxidation states in a strongly acidic medium of high ionic strength), properties, and some therapeutic applications of SG1002 are described in U.S. Patent 8,771,755 (86): The procedure disclosed in this patent yields an impalpable, free-flowing, odorless, fluffy, light yellow microcrystalline powder with the following characteristics: median particle size between 26 and 33 μm, pH of aqueous suspension (0.5 g/50 mL H_2_O) ∼4, solubility in carbon disulfide significantly lower than that of alpha-sulfur, and X-ray diffraction pattern consistent with that of alpha-sulfur. The material used in the clinical trial performed in Australia and synthesized on a large scale under current good manufacturing practices conditions had a bulk density (USP<616>) of 0.6 g/mL; it contained 0.7% residual solvents (0.5% ethanol, 0.2% H_2_O) and 0.4% Na_2_SO_4_.

The inventors of SG1002 attribute its high bioavailability to the hydrophilic nature of the sites on the crystal surface occupied by highly polar moieties such as -SO_3_Na, -S_2_O_2_Na_2_ or -SO_3_H, and they point out that—in striking contrast—the hydrophobic nature of crystals of pure alpha-sulfur is directly related to their low bioavailability (207, 212) and low chemical reactivity in an aqueous medium (116). The small particle size of SG1002 is probably another factor that contributes to its high bioavailability (114).

**FIG. 4.**
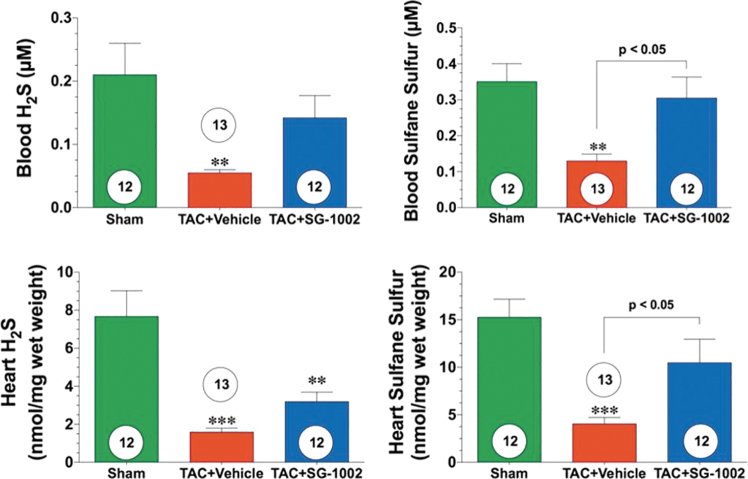
**Heart failure reduces sulfide levels in mice, and SG1002 restores them.** **p < 0.01, ***p < 0.001 *vs.* sham. Reprinted and modified with permission from Kondo *et al.* (134). Copyright 2013 Wolters Kluwer Health, Inc.

Bibli *et al.* (30) found that treatment with SG1002 of human umbilical vein endothelial cells induced protein S-sulfhydration and concomitantly protected membrane lipids from peroxidation; these *in vitro* findings are consistent with the significant increases in H_2_S and/or SS levels of blood and tissues attained when SG1002 is orally administered to mice (134), swine (210), and humans (Fig. 4) (196).

Kondo *et al.* (134), in a study of a murine model of HF, were able to increase levels of circulating H_2_S, circulating SS, cardiac H_2_S, and cardiac SS by about two- to three-fold by orally administering mice 20 mg SG1002/kg/day. Administering the same dose of SG1002 during 12 or 24 weeks to mice fed a high-fat diet (HFD), Barr *et al.* (20) observed increases of between two- and five-fold in H_2_S and SS in the blood and tissue.

**FIG. 5.**
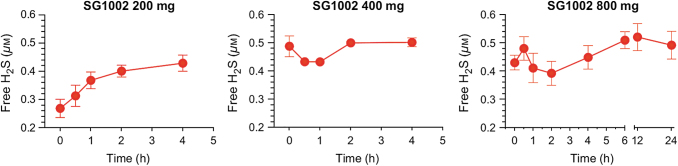
**Healthy subjects: plasma H_2_S (*n* = 5).** Reprinted and modified with permission from Polhemus *et al.* (196). Copyright 2015 John Wiley & Sons, Inc.

In swine that received 800 mg SG1002 “per os” twice a day for 35 days, circulating levels of H_2_S, SS, and nitrite increased by about 3-, 4-, and 2-fold, respectively (210), and in healthy participants in a Phase 1 clinical trial (196), circulating levels of both H_2_S and SS increased by about 2-fold; whereas in HF subjects, the circulating level of H_2_S increased 1.3-fold and that of SS increased about 2-fold (Fig. 5).

**FIG. 6.**
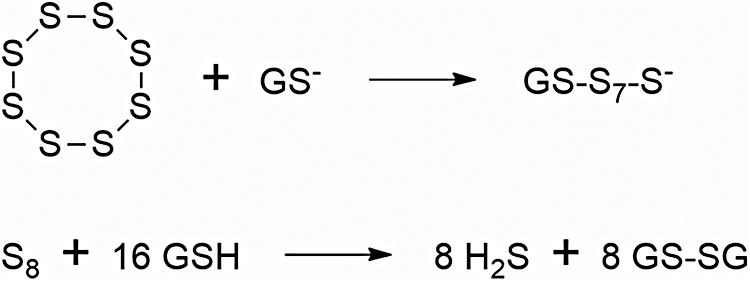
**Mechanism of the reaction between S_8_ and GSH.** GSH, glutathione.

Both the plateauing tendency of the blood H_2_S concentration-*versus*-time curves (31, 196) and the observation of a lack of dependence of Cmax on SG1002 dose (196) are consistent with an overall zeroth-order process comprising chemical reduction of S_8_ to H_2_S in the intestine and H_2_S delivery to the bloodstream. We hypothesize: (i) that the reduction of S_8_ to H_2_S is carried out mostly by intestinal epithelial cells, (ii) that this step involves interaction of a cell's outer membrane (26, 58, 125, 215) with a sulfur species (S_8_, GS-S_7_-S⁻, or HS-S_7_-S⁻) located on the surface of an alpha-sulfur particle (174), (iii) that the high bioavailability of SG1002 is mainly due to the presence of ionic species on the crystal surface (Na_2_SO_4_, Na_2_S_2_O_3_) that render it hydrophilic and/or susceptible to catalysis by GS⁻ or HS⁻ (17, 114, 130, 131, 146, 147, 207, 212), and (iv) that the rate of H_2_S generation is tightly controlled by the cells of the intestinal epithelium (Fig. 6) (33, 34, 82, 180, 206, 217, 260).

Many species of archea, bacteria, and fungi are capable of reducing S_8_ to H_2_S (21, 80, 93, 141, 146, 158, 211), including some species of sulfate-reducing bacteria, and it is highly likely that a large number of members of the intestinal microbiome possess this ability. However, the pharmacokinetic results reported for SG1002 indicate that H_2_S reaches the bloodstream well before SG1002 is delivered to the colon (230).

A series of case studies in terminally ill children aged 18 months to 14 years is presented in the patent just mentioned. These children (all refractory to chemotherapy and/or radiotherapy) presented with osteosarcoma, hydrocephalus with cancerous tumor, medulloblastoma, squamous cell carcinoma, and acute lymphoblastic leukemia; they received between 1200 mg and 3600 mg SG1002 daily with no adverse effects being reported. In all cases, the patient's condition improved—in particular fatigue, inflammation, pain, headache, cardiac function, and glycemia improved—and tumors either shrank or disappeared.

The physicochemical and therapeutic profiles of SG1002 match very closely those of an ideal H_2_S systemic prodrug, since it is:

- Safe, overdose-proof, and with very mild gastrointestinal side effects.
- Orally active.
- Odorless and tasteless (it may be sprinkled on food if the patient has trouble swallowing a capsule or tablet).
- Highly potent.
- Effective independently of H_2_S-generating enzyme levels.
- Able to stimulate enzymatic nitric oxide generation.
- Able to release H_2_S at a slow, constant, and bioregulated rate and to increase SS levels in blood and tissues of all vital organs.
- Indefinitely stable (shelf life greater than 2 years).
- Versatile (highly effective in prevention and/or treatment of multiple pathologies related to inflammation, immune dysregulation, oxidative stress, electrophilic stress, and ER stress).
- Gastroprotective, enteroprotective, hepatoprotective, cardioprotective, renoprotective, neuroprotective, otoprotective, eye protective, chondroprotective, osteoprotective, pancreoprotective, and antifibrotic.

## The Prodrug Approach

### The canonical rational way to develop a drug: the pharmacophore concept

Drug development is a long complex process where a large number of hurdles must be overcome to find and transform a molecule into a chemical entity that has at least the necessary and—preferably—the optimal attributes to be used as a drug for a selected human illness. However, the most fundamental attribute that must be met to start the drug development process is for molecules to interact (bind) with the desired/selected biological target, which, in turn, defines the molecules' mode of action.

Identifying the exact components of a molecule responsible for the interaction (affinity) with a biological target provides a specific set of molecular features that, when present in other molecules, will drive affinity toward the biological target. Examples of molecular features include functional groups, polarity, ionic charges, size, water affinity (hydrophilicity), water repulsion (lipophilicity), and more. The type, position, and distance between themselves further define how these molecular features must be present to effectively enable a molecule to bind to the biological target of interest. These molecular features are referred to as pharmacophores (90). This paradigm, however, is not applicable to the development of multitargeted drugs.

### The classic prodrug concept: classification of prodrugs

One of the many challenges faced in drug development is that potential drug molecules, though potent and selective, suffer from at least a key liability that prevents them from being used as drugs. Examples of such liabilities include poor cell permeability, water solubility, metabolic stability, *etc.*, which will ultimately impact the pharmacokinetic and/or pharmacodynamic properties and profiles of such potential drugs (205).

When faced with these challenges, one strategy used in drug development to overcome such liabilities is to chemically modify a potential drug molecule into a new chemical entity (a prodrug) that, when exposed to specific conditions in an *in vivo* system (*i.e.*, specific pH, chemical reaction, biochemical reaction), will undergo a chemical transformation that will result in forming back the original active molecule/drug (205).

**FIG. 7.**
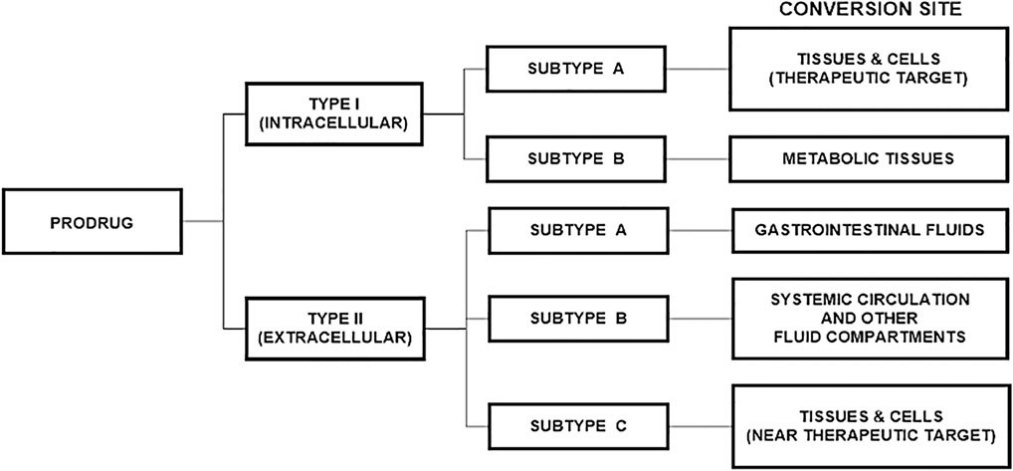
**Prodrug classification.** Adapted from Wu (275).

Prodrugs may be classified on the basis of how they are converted back to the corresponding active compounds. For this, prodrugs are classified into two major categories (Type I, Type II), each with further subcategories based on where prodrugs are converted into active drug molecules *in vivo* (Subtypes IA, IB; Subtypes IIA, IIB, IIC) (Fig. 7) (275).

### Druglikeness, RO5, RO3, Ghose filter, Veber's rule

A 1997 study of the physicochemical properties of Food and Drug Administration (FDA)-approved orally administered drug molecules by Lipinski *et al.* at Pfizer led to the identification of key molecular characteristics that drug molecules share in common. These molecular characteristics together are known as rule of 5 (RO5) and serve as a guidance to determine how drug-like a molecular entity is (druglikeness) (154, 155). The RO5 focuses on what molecular-based properties are to favor the passive absorption/permeability and water solubility of organic molecules; it does not apply to molecules that enter cells *via* active cell-membrane transport, and it does not apply to natural products as well as peptides/peptoids.

In conventional pharmaceutical drug development, guided by the one target-one drug paradigm, a lead compound is a molecular entity that exhibits promising biological activity that also has some good qualities (*i.e.*, physicochemical characteristics) yet its profile can be improved (*i.e.*, cell permeability, water solubility, potency, selectivity). The phase in the drug development process where a lead compound can be further derivatized to overcome limitations (*i.e.*, metabolic instability) and improve its attributes and characteristics (*i.e.*, inhibition potency, biological target selectivity, blood–brain barrier penetration) is called lead optimization.

The RO5 states that oral drugs match at least three of the four following criteria: (i) molecular weight <500 Daltons, (ii) cLogP) <5, (iii) <5 hydrogen-bond donors, and (iv) <10 hydrogen-bond acceptors. However, a recent 2019 study by Shultz at Novartis analyzing small-molecule FDA-approved drugs since Lipinski's study in 1997 revealed that in a 20-year span the molecular weight of FDA-approved drugs went over the suggested 500-Dalton threshold and now has passed the 600-Dalton mark. Moreover, following this trend, the number of hydrogen-bond acceptors has also dramatically increased.

A variation of the RO5 to improve druglikeness prediction is the Ghose filter, which slightly expands the cLogP range (−0.4 to +5.6), further narrows down the molecular weight range (180 to 480 Daltons), and accounts for both molecular refractivity (40 to 130) and number of atoms (20 to 70 atoms, including those participating in hydrogen-bond interactions) (85).

Veber takes a different approach to assess druggability, noting that the 500-Dalton cut-off did not significantly distinguish poorly bioavailable compounds, and that a reduced polar surface area correlates better to permeation over cLogP. Therefore, Veber's rules instead introduce polar surface area (<140 Å^2^) and rotatable bonds (<10) to predict favorable oral bioavailability (for *in vivo* rat models) (256).

During the conventional lead optimization phase of active compounds, generally the molecular weight of the compounds increases, negatively impacting their RO5 profile. Therefore, having active compounds with a “reduced” RO5 profile early in the drug discovery process is advantageous. For this purpose, the RO5 was modified to a rule of three (RO3) to define lead-like compounds. The RO3 parameters are: (i) molecular weight <300 Daltons, (ii) cLogP <3, (iii) <3 hydrogen-bond donors, (iv) <3 hydrogen-bond acceptors, and (v) rotatable bonds <3 (60).

## Development Status of H_2_S Prodrugs

### H_2_S prodrugs in the clinical development stage

Several H_2_S prodrugs have entered clinical trials. Table 2 provides a summary of H_2_S prodrugs in the clinical development stage.

**Table 2.** Clinical Development Status of H_2_S-Releasing Prodrugs

Company	Drug description	Clinical trials	Indication	Bioavailable	Orally active
				S content (% weight)	
Acea Bio (Hangzhou) Co., Ltd.	Fluorapacin (bis(fluorobenzyl) trisulfide)	Phase 1	Advanced cancer	10.17	No
Antibe Therapeutics	ATB-346 (naproxen hybrid)	Phase 1 (completed) Phase 2 (completed) Phase 2 (initiated)	Chronic pain Gastric ulcer Osteoarthritis	8.75	Yes
Bristol-Myers Squibb	Clopidogrel	FDA approved in 1997	Antiplatelet	9.93	Yes
Bristol-Myers Squibb	Zofenopril	FDA approved in 2000	Hypertension; myocardial infarction	7.45	Yes
Gicare Pharma, Inc.	GIC-1001 (trimebutine hybrid)	Phase 1 (completed) Phase 2 (completed)	Pain, cancer, colonic diseases Pain, cancer, colonic diseases	5.30	Yes
Ikaria Therapeutics (Mallinckrodt Pharmaceuticals)	IK-1001 (aqueous sodium sulfide)	Phase 2 (withdrawn) Phase 2 (terminated) Phase 2 (terminated)	ST-segment elevation myocardial infarction Coronary artery bypass surgery Renal impairment	Not applicable	No
Sulfagenix, Inc.	SG1002 (sodium polysulthionate)	Phase 1 (completed) Phase 2 (completed)	Heart failure Male subfertility	99	Yes
PG-PHARMA, LLC	DBTS	Phase 1	Stage IV cancer	11.5	Yes

DBTS, dibenzyl trisulphide; FDA, Food and Drug Administration; H_2_S, hydrogen sulfide.

The development of H_2_S-releasing prodrugs faces many challenges, since “the unique chemical and pharmacological nature of H_2_S necessitates rethinking of some of the general pharmaceutical and drug development principles” (243). For these H_2_S-releasing prodrugs to be pharmacologically effective, they are expected to be water soluble, nontoxic, exhibit a slow *in vivo* metabolic degradation rate, and a slow release of H_2_S *in vivo* (45). Currently, H_2_S prodrugs are being developed by “supramolecular tuning,” that is, as part of drug delivery systems (*e.g.*, polymeric micelles, liposomes, nanoemulsions) (57, 61) that are designed to contain moieties that improve cell permeability and/or targetability (83). Although a handful of these prodrugs have entered clinical trials, most are or have remained in the preclinical development phase, with some of them being used as molecular probes for basic research.

Just as regular drugs, H_2_S-releasing prodrugs can be administered orally (192, 196, 259), intraperitoneally (243), topically (185), ocularly (10), and intravenously (243). The H_2_S-releasing prodrugs that reached clinical trials—ATB-346, IK-1001, GIC-1001, DBTS, and SG1002—are orally administered, with the exception of IK-1001. One of the disadvantages of inorganic sulfide salts, such as IK-1001, is that they rapidly generate H_2_S when in aqueous solution in a pH-dependent manner (243) and volatilization of H_2_S results in lowering of concentration of sulfur species (198), ultimately impacting their handling and biological usefulness.

There are two points worth highlighting regarding the use of SG1002 in preclinical *in vivo* experiments: mode of administration and dosage levels. SG1002 is a versatile H_2_S-releasing prodrug, because it can be given in the food for animals (134, 140) and in tablets or capsules (210). Initially, the dose level of SG1002 for *in vivo* animal testing using mice was set at 20 mg/kg/day (134) and it has remained at this level to date. However, SG1002 Phase 1 clinical trial demonstrated that SG1002 can be administered safely up to 800 mg/day dosages. This has recently prompted the administration of SG1002 in animal studies at higher safe dosages (mice: 307 mg/kg in feed; pigs: 800 mg per tablet) (117, 210) to assess the biological benefits of SG1002, this time at higher safe therapeutic levels going beyond the historical use of SG1002 at suboptimal low dosages.

As can be observed, the reported dosages of SG1002 used in mouse models were 20 and 40 mg/kg/day (117, 134) whereas in the pig model they were 1600 mg/day (800 mg BID) (210). To compare the SG1002 dosages between these two different species and find the equivalent dosage in humans, we conducted an allometric calculation standardizing drug dosage to body surface area according to FDA guidelines (183). Using conversion factors assuming a human body weight of 60 kg and body surface area of 1.62 m^2^, doses of 20 and 40 mg/kg of SG1002 in mouse correspond to 1.6 and 3.2 mg/kg in humans, respectively. For mini-pig allometric calculation, assuming a mini-pig weight of 40 kg, an administration of 1600 mg of SG1002 results in a dosage of 40 mg/kg in the mini-pig, which translates to an equivalent dosage of 36.36 mg/kg in humans. Such a dose of 36.36 mg/kg in humans, and assuming a human weight of 60 kg, translates in administering 2181.6 mg (2.18 g) of SG1002 daily (or 1090.8 mg BID). As shown in the Clinical Studies section [see SG1002 clinical studies: Phase 1 (HF) and Phase 2 (male subfertility) section], for clinical trials a daily dose of 1500 and 1600 mg of SG1002 (750 and 800 mg BID) was chosen, which is 68.75% and 73.3% of the calculated dose using allometric mini-pig parameters. For an individual weighing 60 kg, this would translate to an SG1002 dosage of 25 mg/kg (1500 mg) and 26.67 mg/kg (1600 mg). Now, converting allometrically these human dosages to mouse dosages results in dosages of 351 and 374.5 mg/kg, respectively. This is significant because the majority of, if not all, the reported studies using SG1002 in mouse models have used and continue to use suboptimal dose levels of SG1002 at 20 to 40 mg/kg when, according to these allometric calculations, SG1002 could be used at 9 to 18 times higher levels, which would have an impact on the desired phenotype and *in vivo* therapeutic outcomes.

Table 3 provides a list of patented H_2_S-releasing prodrugs (abandoned patents not included).

**Table 3.** Patented or Patent-Pending H_2_S-Releasing Prodrugs

Assignee	Drug name	Patent number	Status
Antibe Holdings, Inc.	ATB-346	US8541398B2	September 24, 2013 (granted)
Creighton University University of Nebraska University of Houston System		US8092838B2	January 10, 2012 (granted)
Croma-Pharma GmbH		WO2018083326A1	May 11, 2018 (published)
International Society For Drug Development S.R.L.		WO2019129403A1	July 4, 2019 (published)
Istituto Ortopedico Rizzoli, Universita’ Di Pisa		WO2016071863A1	May 12, 2016 (pending)
National University of Singapore	GYY4137	US20100273743A1	September 24, 2013 (granted)
PG-PHARMA LLC	DBTS	US9662303B2	May 30, 2017 (granted)
Sulfagenix, Inc.	SG1002	US8771755B2	July 8, 2014 (granted)
University of Exeter		US10058100B2	August 28, 2018 (granted)
University of New York City Research Foundation		US9688607B2	June 27, 2017 (granted)
University of South Carolina		US20190038643A1	February 7, 2019 (pending)

The production of H_2_S from S_8_ by reaction with GSH is depicted in Figure 6 (207, 240, 241).

### SG1002 as a unique H_2_S prodrug

Among the known H_2_S prodrugs, SG1002 stands out because it lacks a carbon-based scaffold, its properties cannot be tuned or adjusted by conventional lead optimization, it is not a therapeutic targeted-based agent, it is not water soluble, it violates the druglikeness rules (RO5, RO3, Veber's rules), it has a 100% prodrug-to-H_2_S conversion efficiency, and it is bioactivated. The lack of a carbon-based scaffold benefits SG1002 as a therapeutic agent on several fronts. Thus, as the number of atoms in the scaffold increases, a given dose contains decreasing amounts of pharmacophore; further, toxicity is frequently scaffold dependent. Moreover, the fact that SG1002 is orally active presents a huge advantage, because its administration may involve capsules or pills (generally the most accepted and convenient form of administration to patients), which increases patient compliance, avoids painful and intrusive forms of administration such as injections and/or infusions that typically require patients showing up at hospitals/health centers, and a prescheduled time for infusion (*i.e.*, 45 min or longer) all resulting in patients' inconvenience and high rates of noncompliance. Nonetheless, SG1002 is effective in many animal disease models, and it has entered clinical trials where its safety has been demonstrated and preliminary evidence of its efficacy in two different indications has been obtained.

### Preclinical studies of SG1002 in animal models

#### In transverse-aortic-constricted mice (HF model)

SG1002 has been studied in several preclinical disease animal models. Cystathionine gamma-lyase (CSE) is an enzyme that converts L-cysteine into H_2_S, and *in vivo* studies have shown that H_2_S protects against acute myocardial ischemia/reperfusion injury (46). There are also two other enzymes that produce H_2_S, namely, CBS, and 3-mercaptopyruvate sulfotransferase (3-MST). Because SG1002 produces H_2_S, an *in vivo* study was carried out to assess its impact in an HF animal model where myocardial and blood levels of free H_2_S and SS were measured. For the study, SG1002 was administered to C57BL/6J or CSE knockout (KO) mice in the diet to reach a dosage of 20 mg/kg/day and 40 mg/kg/day of SG1002, respectively, 1 week before the animal's thoracic aorta was tied (transverse aortic constriction or transversal aortic constriction [TAC] procedure), with SG1002 dosages given and maintained for up to 12 weeks post-TAC. Moreover, the study also had a separate group of C57BL/6J mice that received SG1002 either 1 or 3 weeks after undergoing the TAC procedure. To determine the expression effects of TAC on CSE, CBS, and 3-MST, the levels of H_2_S in the blood and myocardium were assessed. It was found that CBS expression was not altered, 3-MST expression was downregulated, and CSE expression was upregulated. One aspect of the study included how CSE deficiency impacts heart function after TAC. For this, free H_2_S and SS levels in the heart and blood were compared between CSE KO mice and wild-type (WT) mice. The results show that H_2_S and SS levels were lower in CSE KO mice *versus* WT mice, that CSE KO mice had a much-enlarged heart as well as pulmonary edema *versus* WT mice, and that CSE KO mice exhibited significant left ventricular cavity dilatation. It must be noted that despite these abnormalities, the observed mortality difference between CSE KO mice and WT mice post-TAC was not statistically significant. The results of administration of SG1002 to WT C57BL/6J mice (20 mg/kg/day) post-TAC demonstrate that SG1002 partially restored free H_2_S and significantly restored SS levels in the blood and heart, and that the hearts of SG1002-treated mice showed both significantly less size increase and significantly less pulmonary edema compared with vehicle mice. Moreover, the administration of SG1002 significantly inhibited circulating brain natriuretic peptide (BNP) levels (HF indicator) after TAC. To establish a baseline, SG1002 was administered to CSE KO mice and it was observed that in the blood and heart free H_2_S levels only increased slightly; however, SS levels increased significantly. A salient observation of this study is that administration of SG1002 completely eliminated left ventricular cavity dilatation. Very importantly, this study shows that SG1002 significantly preserved cardiac function after TAC (134).

**FIG. 8.**
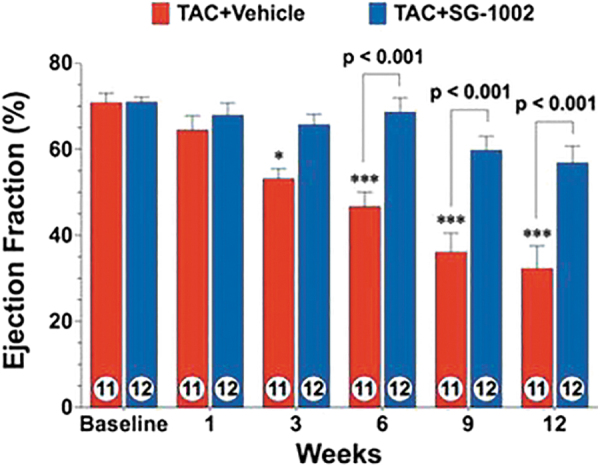
**Ejection fraction from 1 to 12 weeks of TAC.** *p < 0.05, ***p < 0.001 *vs.* baseline. TAC, transversal aortic constriction. Reprinted and modified with permission from Kondo *et al.* (134). Copyright 2013 Wolters Kluwer Health, Inc.

**FIG. 9.**
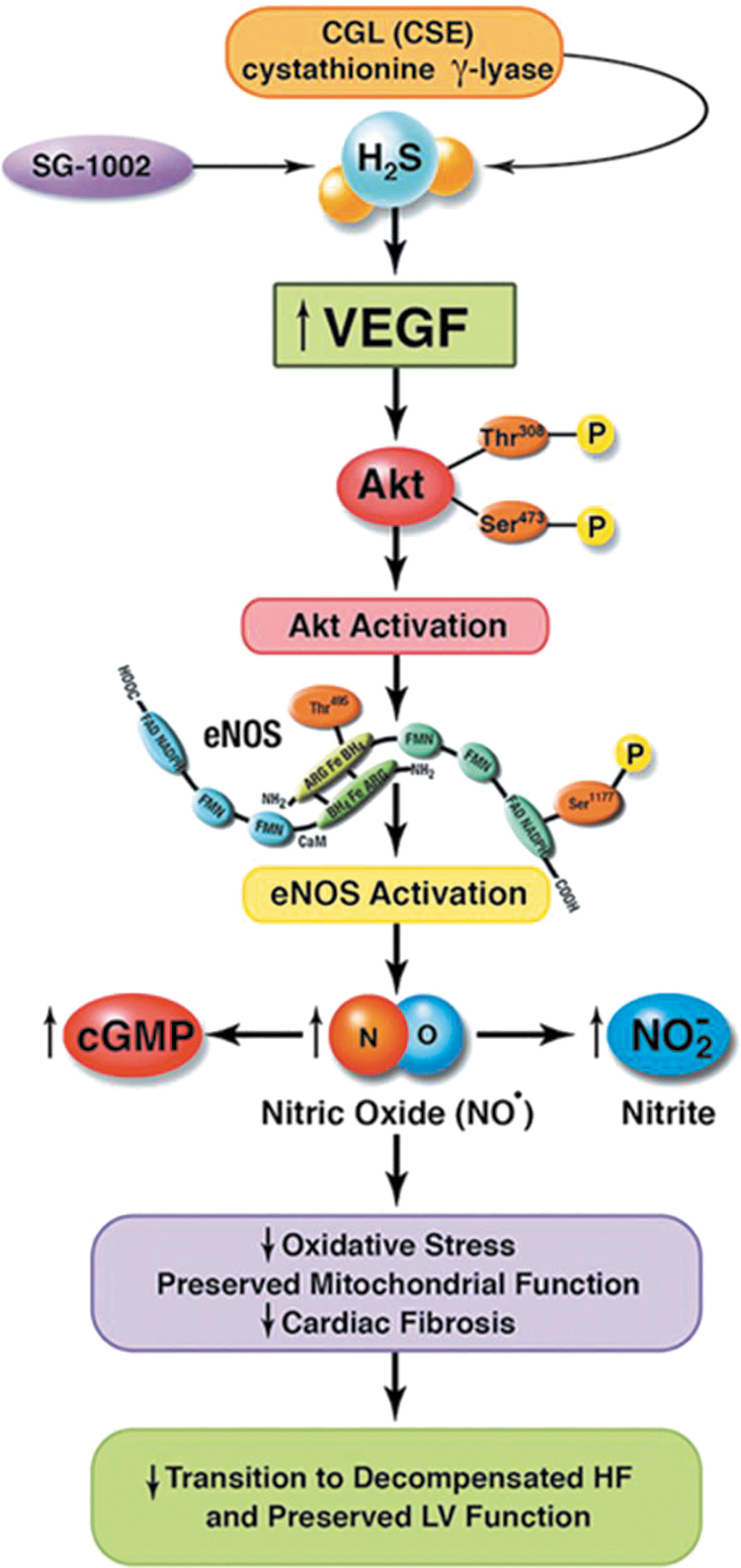
**H_2_S mediated cardioprotection after TAC.** cGMP, current good manufacturing practices; CSE, cystathionine gamma-lyase; eNOS, endothelial nitric oxide synthase; HF, heart failure; LV, left ventricular. Reprinted and modified with permission from Kondo *et al.* (134). Copyright 2013 Wolters Kluwer Health, Inc.

**FIG. 10.**
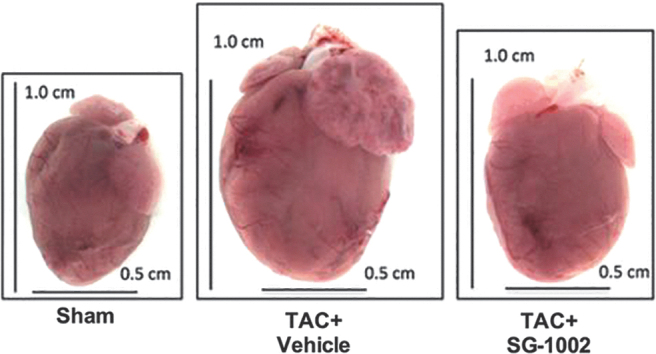
**Representative heart pictures of sham, vehicle-treated (TAC+vehicle), and SG-1002-treated (TAC+SG-1002) mice at 12 weeks of TAC.** Reprinted and modified with permission from Kondo *et al.* (134). Copyright 2013 Wolters Kluwer Health, Inc.

Figures 8–10 provide a summary of the *in vivo* results obtained by using SG1002 in an HF mouse model (134).

#### In an HFD-induced diabetic cardiomyopathy mouse model

**FIG. 11.**
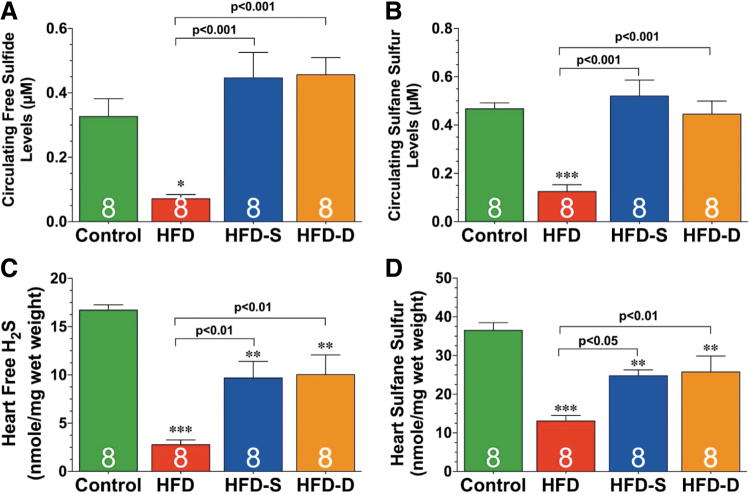
**Circulating (A, B) and cardiac (C, D) levels of free H_2_S and SS.** *p < 0.05, **p < 0.01 and ***p < 0.001 *vs.* control. HFD-D, 60% fat diet; HFD-S, 60% fat diet plus SG1002. Reprinted and modified with permission from Barr *et al.* (20). Copyright 2015 Elsevier B.V.

The SG1002 was also studied in a diabetic cardiomyopathy mouse model where hallmark features of type 2 diabetes and cardiomyopathy were induced by feeding mice an HFD (60% fat) for a period of 24 weeks to assess circulating and cardiac H_2_S levels. The experiment used male C57BL/6J mice divided in three groups: control diet (10% fat), HFD (60% fat), and HFD plus SG1002 20 mg/kg/day. The study found that HFD decreased free H_2_S and SS blood and heart levels. Remarkably, when SG1002 was given to HFD-fed mice, H_2_S blood levels were restored and heart levels partially restored. Again, as mentioned earlier, this was observed while administering low dosage levels of SG1002, which suggests that improved results could be observed if higher safe SG1002 dose levels were used (20). The authors also found that SG1002 therapy restored adiponectin levels and suppressed cardiac ER stress (Fig. 11).

#### In CBS K-O mice

**FIG. 12.**
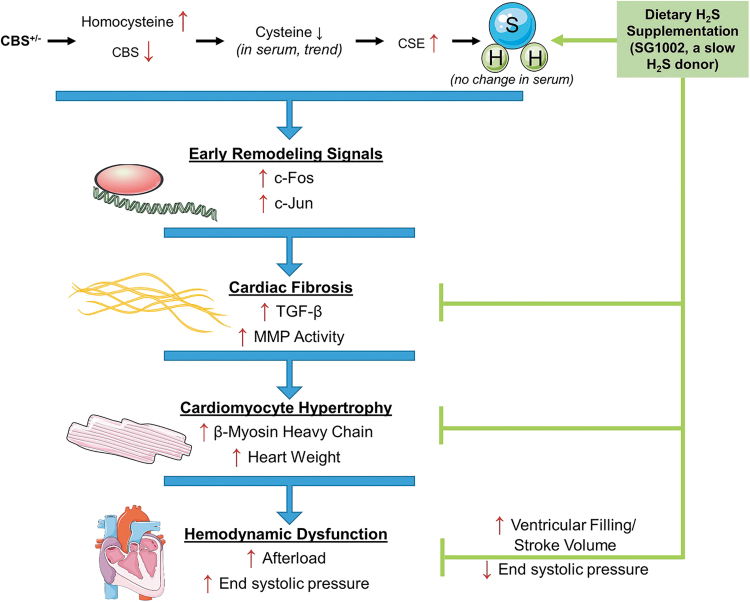
**Working model for the amelioration of cardiac remodeling in HHcy by H_2_S.** CBS, cystathionine beta synthase; HHcy, hyperhomocysteinemia; MMP, matrix metalloprotease; TGF-β, transforming growth factor beta. Reprinted and modified with permission from Kar *et al.* (117). Copyright 2019 Frontiers Media S.A.

A recent study also in the cardiovascular area investigated the effects of SG1002 on homocysteine-induced cardiac remodeling and dysfunction. This 4-month mouse model study used 12-week-old male CBS^+/−^ and sibling CBS^+/+^ (WT); all mice were given SG1002 in food to reach a dose of 40 mg/kg/day. The results demonstrate that CBS^+/−^ mice showed an increased afterload (increased end systolic pressure with conserved stroke volume) and treatment with SG1002 abolished this condition by reducing end systolic pressure and, simultaneously, the end-diastolic volume was significantly increased. The CBS^+/−^ mice developed cardiac remodeling, and SG1002 was found to prevent this unwanted heart condition (Fig. 12) (117).

#### In CSE K-O mice

The research group led by Calvert at Emory University established that restoring H_2_S levels with SG1002 (p.o., 20 mg/kg/day) in the setting of HF increased cardiac mitochondrial content/function, and it improved cardiac function *via* AMPK activation (219).

#### In a porcine model of acute limb ischemia

Another study used SG1002 to assess its proangiogenic effects by using a pig model of acute limb ischemia (ALI). In the 35-day study, pigs underwent the intravascular occlusion procedure to induce ALI and were divided into two groups to receive either placebo or SG1002 (800 mg, oral, BID). The study revealed that pigs that received SG1002 had 2.7 times higher H_2_S circulating levels and 4 times higher level of SS than pigs that received placebo. Moreover, SG1002 also preserved existing capillaries in ischemic limbs to a 1.6 times greater extent than in pigs that received placebo (210).

**FIG. 13.**
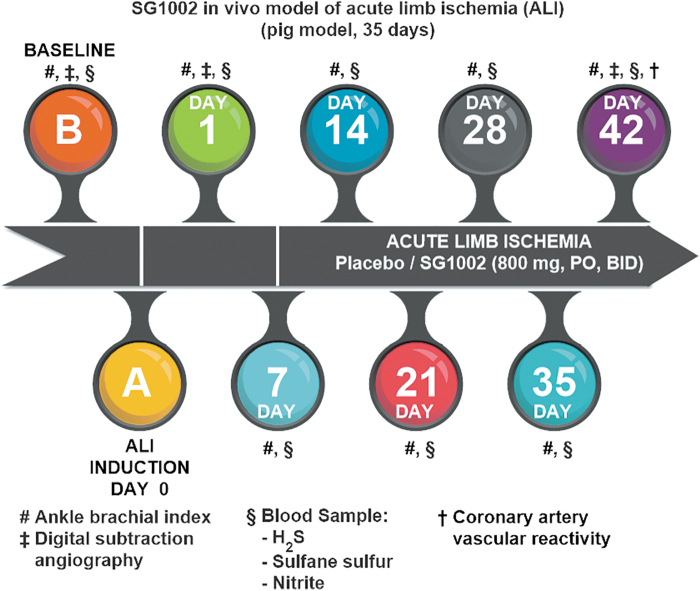
**SG1002 effects in an *in vivo* acute limb ischemia model.** Adapted from Rushing *et al.* (210). Copyright 2019 Elsevier B.V.

Figure 13 depicts the use of SG1002 in an ALI pig model (210).

#### In a murine model of atherosclerosis

A recent study explored the impact of SG1002 against atherosclerosis by using a mouse model. In this 21-day study, the mice underwent surgery and the left carotid artery was partially ligated, with some mice receiving SG1002 (400–600 ng/day). Atherosclerosis was determined by observing differences using immunostaining between ligated left carotid arteries and nonligated right carotid arteries. Mice that received SG1002 showed that plaque formation was significantly reduced, indicating the beneficial antiatherosclerotic effect of SG1002 and its potential therapeutic use for atherosclerosis (31).

### SG1002 clinical studies: Phase 1 (HF) and Phase 2 (male subfertility)

#### SG1002 in HF

A Phase 1 clinical trial was conducted in HF patients to evaluate initial safety and maximum tolerated oral doses of SG1002. Admitted healthy volunteers were 25–34 years old with 19–30 kg/m^2^ body mass index, had a good clinical medical history, and passed a physical examination (*e.g.*, normal electrocardiogram, blood pressure, urine analysis values, heart rate). Congestive HF subjects were between 40 and 71 years old, had symptomatic HF, had a left ventricular ejection fraction of <40%, were able to walk, were class II or III according to the New York Heart Association (NYHA) Classification, normal hemoglobin screening, and had been heart stable for the previous 3 months. Congestive HF subjects having a heart or cardiovascular-related medical incident (*i.e.*, open heart surgery or transient ischemic attack within 3 months of screening, myocardial infarction, hypo- or hypertension), liver disease, infectious disease (*e.g.*, HIV, hepatitis C virus, hepatitis B virus), pregnancy, alcohol abuse, and short life expectancy (<6 months) were excluded from the study. The trial comprised seven healthy subjects (male) and eight HF patients (male, female); enrolled subjects were randomized into two groups (group = one subject placebo: three subjects SG1002) and received placebo or SG1002 orally in escalating dosages (200, 400, 800 mg, BID, each day) for 7 days.

Analysis of free H_2_S levels showed that these levels increased in HF patients at the 400 mg dosage, and in healthy subjects at the 800 mg dosage. In healthy volunteers, H_2_S levels did not exceed 0.7 μ*M* whereas in one HF subject the levels reached 1.1 μ*M*. Curiously, SS levels did not increase significantly in both healthy and HF subjects. On the other hand, nitrite levels significantly increased in both healthy and HF patients when receiving SG1002 at both the 400 and 800 mg dosage level. The BNP levels were obtained at baseline, days 7, 14, and 21 and it was found that patients receiving SG1002 exhibited steady BNP levels at all SG1002 doses. The SG1002 was well tolerated and determined to be safe at all dosages, because only gastrointestinal adverse events were observed (*e.g.*, flatulence, nausea, diarrhea); they were categorized as “mild,” and not all of them were unequivocally proven to be caused by SG1002 (196). Clinical Trial ID: NCT01989208 (https://clinicaltrials.gov/ct2/show/NCT01989208). A Phase 2 trial in HF is planned for 2020.

**FIG. 14.**
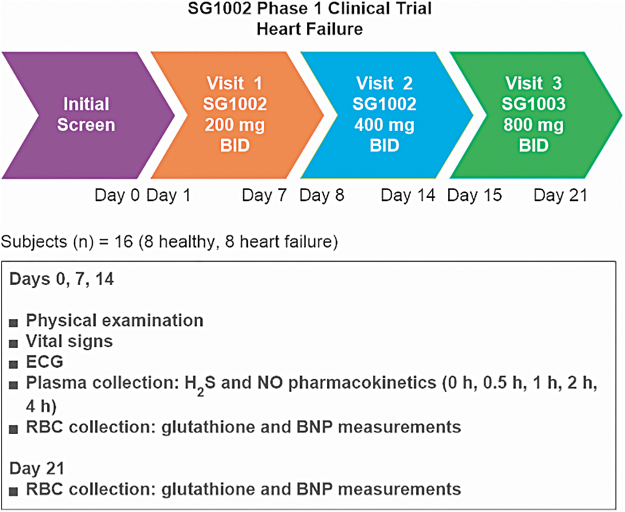
**SG1002 Phase 1 clinical trial.** BNP, brain natriuretic peptide; ECG, electrocardiogram; RBC, red blood cell. Adapted from Polhemus *et al.* (196). Copyright 2015 John Wiley & Sons, Inc.

Figure 14 provides an overview of the SG1002 Phase 1 clinical trial (Clinical Trial ID: NCT01989208) (196).

#### SG1002 in male subfertility

Oxidative stress plays a major role in the etiology of sperm dysfunction *via* induction of peroxidative damage to the plasma membrane. Further, oxidative stress affects the integrity of the sperm nuclear and mitochondrial genomes, leading to DNA strand breaks, aberrant recombination and/or defective packing, as well as chromatin cross-linking (4–8, 23, 47, 59, 87, 123, 162, 167, 187, 223, 252, 294).

The observation of correlations between ROS generation by washed human sperm suspensions and their fertilizing capacity is consistent with the clinical significance of oxidative damage to human spermatozoa; this significance is bolstered by the demonstration of loss of functional competence and high rates of DNA damage of human spermatozoa directly or indirectly exposed to hydrogen peroxide (252).

When the source of ROS is intracellular, many of the classical antioxidants that are effective against extracellular oxidative stress prove useless. However, albumin sustains sperm motility in such instances (252).

As already mentioned, the H_2_S-cysteine-GSH connection suggests that H_2_S may be used by cells to synthesize L-cysteine, which can then serve as a building block in protein synthesis. Sulfur-deficient diets, however, are common and may lead to cysteine deficiency, especially in males, and, consequently, to deficits in the biosynthesis of important cysteine-rich proteins such as so-called CRISPs. The CRISPs are found only in vertebrates, within the male reproductive tract. The CRISPs have been implicated in many aspects of spermatogenesis, as well as in the actual process of fertilization (135), and downregulation of CRISP-2 mRNA by a factor of 4.3 in asthenospermic patients has been reported (112).

The high susceptibility toward irreversible oxidative damage of mammalian sperm cells may be attributed to:

(i) The particularly high content of polyunsaturated fatty acids, plasmalogens, and sphingomyelins of their membranes (4, 6, 47, 59, 123, 167, 294).
(i i) The lack of adequate repair mechanisms for oxidative damage, derived from a dearth of cytosolic antioxidant enzymes associated with the loss of most of their cytoplasm on spermiation (23, 50, 59, 167, 187).
(i ii) Sperm cells are particularly rich in highly active mitochondria, because they need a constant supply of energy to support their motility; in fact, spermatozoa were the first cells found to generate significant levels of ROS (187). These characteristics increase the probability of mitochondrial membrane damage by leaked ROS.
(i v) Native CRISPs present unusually high numbers of thiolic (unoxidized) cysteine residues, which renders them especially sensitive to inactivation by oxidants.

A randomized controlled Phase 2 trial was performed by using SG1002 on men presenting with idiopathic oligoasthenozoospermia. In the 75-day study, 54 subjects with oligoasthenozoospermia were randomly divided into three cohorts (one cohort = 18 men), administered resveratrol (25 mg +725 mg microcrystalline cellulose), SG1002 (750 mg capsule), and placebo (725 mg microcrystalline cellulose); sperm analysis was performed at the beginning and end of treatment. The results showed that sperm concentration did not change for men in the placebo and resveratrol cohorts; however, men administered SG1002 exhibited higher sperm concentration and sperm motility and a lower percentage of morphologically abnormal spermatozoa *versus* men in the placebo cohort. The results not only demonstrate the benefits of using SG1002 against idiopathic oligoasthenozoospermia but also further confirm the safety profile of SG1002 at a dosage of 750 mg/day when administered daily during a period of 75 consecutive days (Table 4) (178).

**Table 4.** Clinical Trial: Effect of SG1002 on Male Subfertility

Methods	Double-blind, placebo-controlled randomized trial Allocation concealment: Numbered bottles delivered to the site with all members of the trial blinded to numerical code.
Participants	Country: Mexico (State University of Nuevo León, UANL, Monterrey) Infertile men recruited from UANL's Reproductive Biology Clinic at the University Hospital. Mean age: 34.23 (range 24–42). *n* = 44 men completed the study. Inclusion criteria: Age 20–45 years/regular sexual intercourse with a potentially fertile female/with diagnosis of infertility (oligozoospermia and/or asthenozoospermia)/normal motility <50% Sperm count between 5 × 10^6^/mL and 20 × 10^6^/mL. Exclusion criteria: Sudden improvement or decrease in semen quality during run-in/presence of serious disease (diabetes, CVD, *etc.*)/treatment with antioxidants within 6 months of recruitment/evidence of tobacco, alcohol, or drug addiction. Duration of the study: 15 months.
Interventions	SG1002 (a proprietary H_2_S prodrug): 1500 mg/day (*n* = 16) *versus* Resveratrol: 50 mg/day (*n* = 14) *versus* Microcrystalline cellulose (placebo) (*n* = 14) Duration of treatment: 75 days
Outcomes	Primary: semen parameters Secondary: pregnancy rate
Notes	The study was carried out between July 2009 and September 2010. 72 patients were recruited originally. 18 patients were excluded, because they did not satisfy the inclusion criteria. 1 patient abandoned the study, because “his perspiration had a strange smell.” 1 patient abandoned the study after 3 days of treatment, on complaining of nausea/flatulence. 1 patient abandoned the study, because “the course of treatment involved taking too many capsules per day.” 7 patients presumably completed the treatment but were lost to follow-up.

There were no significant differences between the different treatment groups in terms of important baseline parameters.

The outcomes for the group receiving SG1002 were as follows:

- The percentage of morphologically abnormal spermatozoa decreased from 60% to 36% (*p* = 0.017).
- The percentage of still spermatozoa decreased from 63% to 27% (*p* = 0.039).
- Sperm concentration increased from 10.32 × 106/mL to 17.73 × 106/mL; however, this difference was not statistically significant.

The limited number of participants in this study and the favorable tendency in the sperm counts make it highly likely that a statistically significant increase in this endpoint will be observed in a full-fledged higher-powered clinical trial.

These results should be considered highly encouraging, especially against a backdrop of marginally effective therapeutic options for male subfertility, which will be briefly discussed next.

In the three Cochrane reviews on “Antioxidants for male subfertility” (221, 222, 228), the authors assessed the effects of oral antioxidants on men with documented sperm DNA damage and/or with impaired semen parameters on the basis of clinical trials wherein the participants were randomly assigned to antioxidant *versus* placebo, an alternative antioxidant, or no treatment; they found that, overall, the current evidence is inconclusive.

This means that there is only limited scientifically acceptable evidence that antioxidant supplementation improves outcomes for subfertile couples or—in Agarwal's words—that “the available forms of treatment have mostly produced only marginally satisfactory responses, even in the best of proper trials” (6) and that many drugs are being used without any rationale. On the other hand, the recently published results of Wang *et al.* (262) validate H_2_S supplementation as a scientifically sound approach to treatment of oxidative stress-related male subfertility.

One final consideration is warranted: Semen quality is a marker not only of fecundity but also of general health; impaired semen quality has been associated with shorter life expectancy and enhanced long-term morbidity (76, 77, 108, 144). Therefore, the positive influence of SG1002 on semen quality is consistent with the effects of H_2_S on general health and lifespan.

## Conclusions

DATS, DBTS, TC-2153, and SG1002 have the potential to become safe and effective pharmacological therapeutic agents that collectively will prove to be invaluable in humanity's fight against the ravages of hundreds of disease conditions related to oxidative stress and cellular damage inflicted by ROS: These conditions include most aging-related diseases. This potential is, in the case of SG1002, exclusively based on the fact that it is an H_2_S prodrug, whereas the other donors elicit pharmacologic effects that are only partially mediated by H_2_S.

To realize the therapeutic potential of these four agents, it will be necessary to invest considerable resources to carry out the required clinical trials, since—to the best of our knowledge—only in one case (SG1002) has safety been demonstrated in a formal Phase 1 clinical study.

For SG1002, mode-of-action studies will be required to determine the locus and mechanism of H_2_S production and absorption; it will be necessary—for example—to validate the hypothesis of chemical reduction of S_8_ to H_2_S by cells of the intestinal epithelium and to study a possible participation of the intestinal microbiome.

DATS, DBTS, and SG1002 also hold the promise of becoming exceptionally useful prophylactic and antiaging agents that are capable of promoting immunity, helping to either prevent or retard the onset of chronic-degenerative diseases and to protect the vital organs against damage induced by paracetamol, corticosteroids, NSAIDs, anticancer drugs, *etc.* SG1002 has an advantage in this context, namely its clean quantitative conversion into H_2_S without causing ill-smelling breath and body odor.

SG1002 is emerging as a leading H_2_S donor on account of its safety, mode of administration, and unique ability to efficiently generate H_2_S with no byproducts in a slow and sustained manner that is dose independent and enzyme independent. These features position SG1002 as the H_2_S donor of choice when studying biological systems *in vivo*, whereas its almost nil water solubility makes it the least attractive one to choose for *in vitro* experiments.

Novel sulfur-rich, safe, and effective LMW H_2_S/SS donors for specific purposes may be designed, synthesized, characterized, and developed in a rational fashion and guided by the massive knowledge now available.

Efficient tuning of H_2_S donation characteristics (*e.g.*, release rate, tissue targeting, *etc.*) may be achieved not only by conventional systematic structural modification of a lead molecule but also through “supramolecular tuning,” as in the cases of SG1002 and of DATS-loaded polymeric nanoparticles.

## Abbreviations Used

3-MST: 3-mercaptopyruvate sulfotransferase
4-HNE: 4-hydroxy-2-nonenal
ACE: angiotensin converting enzyme
AD: Alzheimer's disease
AIDS: acquired immunodeficiency syndrome
ALI: acute limb ischemia
BID: two each day
Bn: benzyl
BNP: brain natriuretic peptide
BnSAc: benzyl thioacetate (S-acetyl benzylmercaptan)
BnSH: benzyl mercaptan
CBS: cystathionine beta synthase
cGMP: current good manufacturing practices
cLogP: calculated logarithm of the octanol-water partition coefficient
Cmax: maximal serum concentration
CRISP: cysteine-rich secretory protein
CSE: cystathionine gamma-lyase
Cys: L-cysteine
DADS: diallyl disulfide
DAS: diallyl sulfide (diallylthioether)
DATS: diallyl trisulfide
DATTS: diallyl tetrasulfide
DBDS: dibenzyl disulfide
DBTS: dibenzyl trisulfide
DBTTS: dibenzyl tetrasulfide
ECG: electrocardiogram
eNOS: endothelial nitric oxide synthase
ER: endoplasmic reticulum
FDA: Food and Drug Administration
GSH: glutathione
GSSG: glutathione disulfide
H_2_S: hydrogen sulfide
H_2_S_2_: hydrogen persulfide
HD: Huntington's disease
HF: heart failure
HFD: high-fat diet
HFD-D: 60% fat diet
HFD-S: 60% fat diet plus SG1002
HHcy: hyperhomocysteinemia
HIV: human immunodeficiency virus
HO-1: heme oxygenase-1
HSA: human serum albumin
IC50: half maximal inhibitory concentration
ip: intraperitoneal
KO: knockout
LD50: median lethal dose
LG: leaving group
LMW: low-molecular-weight
LogP: logarithm of the octanol-water partition coefficient
LV: left ventricular
ME: 2-mercaptoethanol
MMP: matrix metalloprotease
Nrf2: nuclear erythroid 2-related factor 2
NYHA: New York Heart Association
OSC: organic sulfur compound
PD: Parkinson's disease
pKa: Log(1/(acidity constant))
Pr-SH: protein with a free thiol group
RBC: red blood cell
RDS: rate-determining step
RO3: rule of three
RO5: Lipinski's rule of five
ROS: reactive oxygen species
SS: sulfane sulfur
STAT3: signal transducer and activator of transcription 3
STEP: striatal-enriched protein tyrosine phosphatase
TAC: transversal aortic constriction
TGF-β: transforming growth factor beta
WT: wild-type

## Appendix

### Barriers to Clear Thinking on Sulfane Sulfur and Hydrogen Sulfide

Valence and oxidation number of sulfane sulfur (SS) atoms in a molecule (Appendix Fig. A1).

1. SS atoms are not zerovalent; since they are attached to another sulfur through a double bond (as the “outer” sulfur in thiosulfate, thiosulfinates, and thiosulfonates) or—more commonly—to two other atoms by single bonds, they are divalent. They may be found attached to either two sulfur atoms (*i.e.*, the central sulfur in diallyl trisulfide) or a sulfur and a hydrogen atom—for instance the sulfur atoms in HS-SH (102, 253).

2. The formal oxidation number (193) assigned to an SS atom is either minus one (as in HS-SH) or zero—as all atoms in S_8_ or the “outer” S atom in thiosulfate.

Please note that all atoms in S_8_ are divalent; however, in chemistry and many scientific disciplines (*e.g.*, biochemistry, geochemistry, biology, limnology, oceanography, *etc.*), S_8_ is often mistakenly assumed to comprise zerovalent sulfur.

3. SS is not “bound sulfide,” since the oxidation number of S in sulfides (including hydrogen sulfide [H_2_S]) is minus two. The expression “bound sulfide” is extremely misleading, because an SS atom, which is always joined to at least another sulfur atom, is not in the minus two oxidation state and because the conversion of H_2_S into SS is a redox process that, consequently, requires the intervention of an oxidizing agent.

#### SS defined

1. Most recent scientific papers offer unsatisfactory definitions of SS that are usually either over-restrictive or too inclusive. The SS is definitely not “a sulfur atom with six valence electrons and no charge,” a definition that is—unfortunately—gaining popularity in the scientific literature. This definition should be discarded for four reasons: (i) It wrongly includes or seems to include all divalent sulfur atoms; (ii) it wrongly excludes or seems to exclude the “outer” S atoms of deprotonated polysulfanes and hydropolysulfides; (iii) it misses the essential requirement that an SS atom be always attached to another sulfur atom (i.e., all SS atoms are catenated); and (iv) it is not clear whether “charge” refers to actual electric charge (as on the S atom of a thiolate anion) or formal charge or oxidation number (193).

2. Operationally, SS was long ago defined (273) as “cyanolyzable sulfur,” that is, sulfur that reacts at pH 8.5–10 with a cyanide ion to yield a thiocyanate ion, and in fact all divalent sulfur atoms in the zero or minus one oxidation state and attached to at least one S atom or to S and H conform to the said definition. However, sulfur atoms in the minus one oxidation state that are attached to divalent sulfur and to an “activating group” such as allyl, benzyl, or phenacyl also yield thiocyanate on cyanolysis, and they have traditionally been regarded as SS as well (Appendix Fig. A2) (101, 102, 273).

Thus, one of the sulfur atoms in diallyl disulfide (DADS) is susceptible to conversion into thiocyanate on cyanolysis:

$$\begin{array}{cc} CH_{2} & =CH-CH_{2}-S-S-CH_{2}-CH\\ & =CH_{2}+CN\to CH_{2}=CH-CH_{2}-CN+CH_{2}\\ & =CH-CH_{2}-S-SCH_{2}\\ & =CH-CH_{2}-S-S+CN\to CH_{2}\\ & =CH-CH_{2}-S+NCS \end{array}$$

Hence, DADS belongs to the “SS pool” according to the operational definition; the carbon–carbon double bond electronically “activates” DADS and renders it susceptible to nucleophilic substitution on either of its alpha carbon atoms.

3. In summary, we propose the following definition: An SS atom is divalent, catenated, and in a 0 (zero) or −1 formal oxidation state; if in the latter, it must be attached to either an H atom or an “activating group” such as allyl, benzyl, or phenacyl. Possible exceptions to this generalization are compounds whose molecules comprise selenium-sulfur bonds and metal-sulfur bonds; to circumvent this limitation, the expression “a H atom” may be replaced by “an atom less electronegative than sulfur.”

#### Not all SS species are equally reactive

1. Disulfane, polysulfanes, hydropersulfides, hydropolysulfides, organic polysulfides, and higher sulfanedisulfonic acids (polythionates) react quantitatively with cyanide in alkaline solution at room temperature (*i.e.*, under “cold cyanolysis” conditions); whereas thiosulfate and trithionate require “hot cyanolysis” (273). DADS has been reported (101) to react sluggishly under “cold cyanolysis” conditions. To the best of our knowledge, it is not known whether it reacts quantitatively under “hot cyanolysis” conditions; this sluggishness is consistent with reports on its low reactivity toward thiols (43, 153), but not with the earlier results of Benavides *et al.* (26), which were probably flawed by the presence of about 10% DATS in the lot of DADS used by these authors.

Dissolved elemental sulfur, colloidally dispersed elemental sulfur, and polysulfanes in aquatic samples react quantitatively under slightly acidic “hot cyanolysis” conditions, whereas nondissolved and noncolloidal particulate elemental sulfur (*i.e.*, solid alpha-sulfur) does not react under such conditions (115). This particle-size-dependent reactivity of S_8_ (colloidal particles > larger particles) is consistent with the higher surface free energy and specific surface area of the S_8_ colloids (233).

Elemental sulfur (S_8_) is commonly found in nature as crystalline alpha-sulfur; it is highly hydrophobic and its aqueous solubility (115) is extremely low (about 19 n*M*): Importantly, the availability of alpha-sulfur to both sulfur-reducing and sulfur-oxidizing bacteria depends critically on their ability to overcome this “hydrophobic barrier.” On account of the high variability of particle size and surface properties, the reactivity and bioavailability of particulate alpha-sulfur show a wide range of values differing by factors as high or higher than 100.

#### H_2_S, HS⁻, and the biological SS pool

In the absence of oxidizing agents, aqueous solutions of H_2_S comprise only two sulfur-containing chemical species (H_2_S and HS⁻) in dynamic acid-base equilibrium. It should be noted that, on account of their very high basicity, sulfide ions are never present in aqueous media (172).

As soon as molecular oxygen—or a biologically relevant oxidant—enters the scene, a myriad of inorganic and organics SS species are generated and many participate in both acid-base and redox equilibria. Therefore, it becomes quite difficult to decide, in a given context, whether the specific biological effector is H_2_S, HS⁻, or an SS species. In some cases, however, we may *a priori* rule out one or more species on the basis of basic chemical or thermodynamic considerations: For example, direct oxidative activation of nuclear transcription factor Nrf2 by H_2_S (or HS⁻) is not possible, simply because H_2_S and HS⁻ comprise S in the −2 oxidation state (the lowest possible one) and therefore can act only as reductants.

#### H_2_S misrepresentation and a recommendation

1. Another barrier to clear thinking that permeates the biomedical literature, namely the unjustified and almost obsessive conceptualization of H_2_S as a gas, is evident in the frequent use of expressions such as “H_2_S gas” or “the gaseous nature of H_2_S” or in the representation of H_2_S as bubbles, notwithstanding the fact that H_2_S is present in a condensed phase in the overwhelming majority of tissues and organs—namely in liquid solution in an aqueous phase.

Any given molecule is not inherently gaseous, liquid, or solid, and it is hard to understand how clarity may be served when an author “uses the term H_2_S only in specific reference to the gas, and in all other instances sulfide will refer to combinations of the gas and the anion,” or states that “in this article, the sum of both molecular forms (H_2_S and HS⁻) will be referred to as H_2_S, whereas the term H_2_S will be reserved for the diprotonated, volatile form” or equivocally writes that “it may be possible to store or transport sulfide in a less labile, nongaseous state.” These statements—and many others found in currently published articles—attest to the dangers of univocally associating a molecule with a state of matter.

2. We believe that terminology and notation are important and should be tools that facilitate thought and enable an idea to be expressed with precision. Significant progress in science often has been made after an appropriate notation, which allows for an accurate, compact, and unambiguous description, is introduced (255). The histories of mathematics and physical science provide excellent examples of the importance of good notation, including the invention of the Arabic notation in mathematics, the creation by Dalton of the atomic symbolism in chemistry, and Linnaeus' introduction of the binomial nomenclature in biology (99).

Hence, we earnestly recommend that high priority be assigned to the task of generating agreement on terminology and crafting a notation that will allow precise and compact descriptions of aqueous systems comprising H_2_S, HS⁻, and SS, and will facilitate their understanding instead of hindering it.
